# Protein S-sulfenylation is a fleeting molecular switch that regulates non-enzymatic oxidative folding

**DOI:** 10.1038/ncomms12490

**Published:** 2016-08-22

**Authors:** Amy E. M. Beedle, Steven Lynham, Sergi Garcia-Manyes

**Affiliations:** 1 Department of Physics and Randall Division of Cell and Molecular Biophysics, King's College London, London WC2R 2LS, UK; 2 Centre of Excellence for Mass Spectrometry, King's College London, London SE5 8AF, UK

## Abstract

The post-translational modification S-sulfenylation functions as a key sensor of oxidative stress. Yet the dynamics of sulfenic acid in proteins remains largely elusive due to its fleeting nature. Here we use single-molecule force-clamp spectroscopy and mass spectrometry to directly capture the reactivity of an individual sulfenic acid embedded within the core of a single Ig domain of the titin protein. Our results demonstrate that sulfenic acid is a crucial short-lived intermediate that dictates the protein's fate in a conformation-dependent manner. When exposed to the solution, sulfenic acid rapidly undergoes further chemical modification, leading to irreversible protein misfolding; when cryptic in the protein's microenvironment, it readily condenses with a neighbouring thiol to create a protective disulfide bond, which assists the functional folding of the protein. This mechanism for non-enzymatic oxidative folding provides a plausible explanation for redox-modulated stiffness of proteins that are physiologically exposed to mechanical forces, such as cardiac titin.

## Abstract (web summary)

Protein S-sulfenylation is a posttranslational modification that can act as a sensor of redox oxidative stress. Here the authors show that, following mechanical unfolding, sulfenic acid drives disulfide bond reformation and guides non-enzymatic oxidative folding.

## Graphical abstract

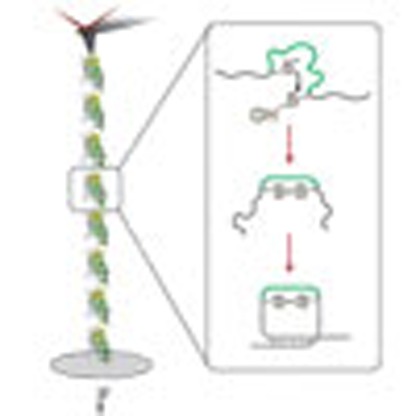

Protein folding defines a molecular self-assembly process that is finely tuned by a subtle interplay between enthalpic and entropic contributions^1^,^2^,^3^. An extra layer of complexity is added when a native covalent disulfide bond needs to reform along the folding pathway^4^,^5^. Other chemical alterations, such as post-translational modifications, pose further challenges to the successful completion of the folding process.

Cysteine is a principal target for post-translational modifications, especially those related to oxidative stress^6^. Due to the presence of empty *d*-orbitals ready for bonding, the highly polarizable sulfur atom can display oxidation states ranging from −2 to +6 (refs 7, 8). Cysteine sulfenic acid (Cys-SOH) is the first oxidation step of cysteinyl thiols (Cys-SH)^9^ and the resulting post-translational modification, cysteine S-sulfenylation, has been identified as a redox sensor in an increasing number of proteins^10^,^11^. However, the unusually high reactivity of -SOH—a consequence of its dual electrophilic and nucleophilic character^7^,^10^,^12^—, together with its invisibility to ultraviolet–visible and fluorescent spectroscopic detection^13^, has rendered its characterization challenging. In general, stabilization of protein sulfenic acid is highly dependent on the protein microenvironment^11^, following the general notion that solvent-exposed cysteines feature increased chemical lability^14^.

Identification of protein sulfenic acids has been mostly indirect, through chemical labelling with 5,5-dimethyl-1,3-cyclohexadione (dimedone)^15^, a mass-shifting molecular probe that specifically traps -SOH moieties and is easily identifiable with mass spectrometry (MS)^16^,^17^,^18^. Despite this phenomenal progress^12^, given the irreversible nature of the detection methods, which interfere with the intrinsically fast reactivity of Cys-SOH, direct identification of the dynamics of a particular protein -SOH and its implication on folding remains largely elusive.

Under oxidative conditions, Cys-SOH can be readily oxidized to more stable products, namely sulfinic (Cys-SO_2_H) or sulfonic (Cys-SO_3_H) acids^6^. The transition to these higher oxidation state species has been generally regarded as irreversible, and a marker for oxidative damage. Such deleterious pathway can be avoided if the electrophilic sulfur atom in -SOH reacts with an intramolecular neighbouring protein thiol nucleophile to form a stable disulfide bond^19^,^20^. Such oxidant-mediated disulfide bond formation process, typically occurring within the reducing conditions of the cytosol on a transient increase in oxidative stress, differs from the enzyme-catalysed formation of structural disulfide bonds. This latter process takes place in the oxidizing milieu of the endoplasmic reticulum or the mitochondrial inter membrane space in eukaryotes or in the periplasmic space in bacteria^19^,^21^ via the formation of a mixed disulfide intermediate between the catalytic enzyme and the protein substrate^22^,^23^. Despite its *in vivo* relevance, a comprehensive molecular description of the formation of disulfide bonds within the context of non-enzymatic oxidative folding is still missing.

Here we use a combination of single-molecule force-clamp spectroscopy, protein engineering techniques and MS to directly monitor the reactivity of an individual force-induced -SOH moiety occurring within a single immunoglobulin domain of the giant titin protein. Our results demonstrate that the fleeting -SOH intermediate, the life-time of which is largely conformation dependent, acts as a molecular switch that directly modulates protein function. In a short window of opportunity in the second timescale, the -SOH moiety is able to avoid irreversible protein misfolding—induced by cysteine hyperoxidation and/or aldehyde condensation—by readily forming a disulfide bond that guarantees the functional final folding of the protein. We hypothesize that this mechanism for non-enzymatic oxidative folding may be a common place for oxidation-induced post-translational modifications occurring on solvent-exposed cysteines, drastically affecting the elastic properties of proteins that are exposed to mechanical forces.

## Results

### -SOH triggers disulfide bond formation

**Figure 1:**
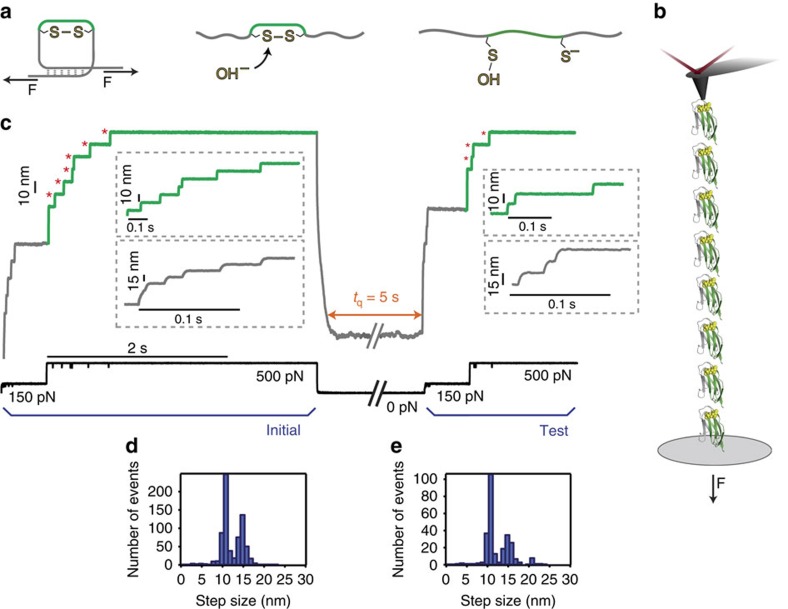
Single-molecule force-clamp spectroscopy captures the non-enzymatic reformation of individual disulfide bonds. (**a**) The hydrolysis of the protein disulfide bond via a S_N_2 attack by hydroxide anions results in the creation of a sulfenic acid and thiolate. (**b**) Schematics of the engineered (I27_E24C–K55C_)_8_ polyprotein being stretched in the AFM set-up. (**c**) Pulling on an individual polyprotein at constant force using a specifically designed pulse-protocol captures oxidative protein folding. The initial pulse unfolds the protein and ruptures the disulfide bond; the first pulse *F*=150 pN unfolds and extends all the modules of the (I27_E24C–K55C_)_8_ polyprotein to the mechanical clamp created by the disulfide bonds, which exposes them to the solution (grey line). Each unfolding event is marked by a step increase of ∼15 nm (inset, grey line). A second stronger force pulse (*F*=500 pN) triggers disulfide rupture (green line), fingerprinted by equally spaced ∼10 nm steps (inset, green line) that correspond to the extension of the peptide chain released on the scission of each disulfide bond (red asterisks). Releasing the pulling force (*F*=0 pN) triggers the protein to collapse and fold for a time quench *t*_q_=5 s. The subsequent test pulse probes the folding status of the protein. Mirroring the initial pulse, a force pulse of *F*=150 pN unfolds and extends the refolded protein up to the newly created disulfide bond (inset, grey line) whereas the last *F*=500 pN pulse is able to rupture it again (inset, green line). The step-size histograms corresponding to the (**d**) initial and (**e**) test pulse highlight in both cases the presence of ∼10 nm and ∼15 nm steps.

-SOH is typically induced by exposing a cysteine residue to high concentrations of hydrogen peroxide (H_2_O_2_)^24^. However, its fast kinetics (10–10^7^ M^−1^ s^−1^)^25^,^26^ and its further reactivity to higher oxidation state species often precludes the capture of this transient -SOH intermediate. An alternative means to create a protein -SOH in a much more controlled manner is based on the alkaline hydrolysis of an individual disulfide bond, resulting in the stoichiometric formation of a -SOH and a thiolate^27^,^28^,^29^ (Fig. 1a). Albeit admittedly less physiologically relevant, this strategy is also found in nature^30^,^31^, as reported for phosphoglycerate kinase reductase^32^,^33^.

Using an experimental single-molecule mechanical assay designed previously^34^, an individual -SOH moiety can be readily created by the hydrolysis of a single protein disulfide bond under high-pH conditions (Fig. 1a). The atomistic detail of this process has been provided by highly revealing molecular quantum mechanics/molecular mechanics (QM/MM) simulations^35^,^36^. We now set out to extend our experimental approach to probe whether each cleaved disulfide bond can be reformed in the absence of a catalytic enzyme, and how such putative reformation process affects the entire protein folding phenomena.

In our experiments, we used a single-molecule force-clamp atomic force (AFM) spectrometer (Fig. 1b) to stretch under high alkali conditions (pH=12.8), a polyprotein containing eight identical domains of a mutated titin Ig27 domain containing two cysteines in positions 24 and 55, which readily form a structurally buried disulfide bond (Fig. 1a). We designed a five-pulse force protocol to measure the protein elongation over time, which enables independent capture of each individual cleavage and reformation event, with single bond resolution (Fig. 1c). Applying an initial pulse of 150 pN for 0.5 s to the (I27_E24C–K55C_)_8_ polyprotein resulted first in the presence of fast elongation steps of ∼15 nm (inset, grey), corresponding to the unfolding and extension of each individual protein domain up to the rigid disulfide bond, which became exposed to the solvent environment (Fig. 1c, grey). A second force pulse at a much higher force (500 pN) triggered the forced-induced scission of each individual disulfide bond via a S_N_2 nucleophilic attack by the hydroxyl (OH^−^) anion. Each individual hydrolysis event is fingerprinted by a step of ∼10 nm (inset, green), corresponding to the extension of the amino acids that were trapped behind the disulfide bridge (Fig. 1c, green). The two distinctive step-size populations in these first two high-force pulses (initial pulse) are clearly observed in the bimodal histogram shown in Fig. 1d. Notably, after the cleavage of all disulfide bonds at 500 pN, the fully unfolded and extended polyprotein is extended to almost its contour length and all of its backbone residues are fully exposed to the chemical environment of the measuring solution.

Shortly after the last individual rupture event, the stretching force was completely withdrawn for *t*_q_=5 s to trigger the folding of the polypeptide. Such force-quench approach allows dissection of the distinct phases involving mechanical folding from highly extended states^37^,^38^,^39^,^40^. After the quenching pulse, a test pulse—composed again of a stretching sequence of 0.5 s at 150 pN followed by a pulse at a higher force of 500 pN— was applied to the protein to probe its folding status.

The elongation pattern of the protein during the test pulse enables discrimination among three different possible oxidative folding scenarios; (i) if the protein has not been able to fold within the quench time, it will extend featuring the absence of well-defined steps^37^,^39^. Instead, (ii) if the protein is able to refold—thus regaining mechanical stability—but the disulfide bond has not been reformed, the protein will extend featuring steps of 25 nm, corresponding to the unfolding and stretching of the fully folded protein in the absence of the mechanically rigid, covalent disulfide bond^38^. Finally, (iii) if the protein has been able to both successfully refold and reform the disulfide bond, the test pulse will be then composed of the unfolding ∼15 nm steps followed by the ∼10 nm steps corresponding to the (re)-rupture of each successfully reformed disulfide bond. In summary, a 25 nm step provides the signature of a properly folded domain with no disulfide, whereas a 15 nm step (followed by a 10 nm step) indicates a properly folded domain with a formed disulfide.

The latter scenario was indeed observed in our folding trajectories (Fig. 1c). The histogram corresponding to the distribution of step sizes populating the test pulse hallmarks the presence of both ∼15 nm and ∼10 nm steps (Fig. 1e). Surprisingly, not a single event corresponding to the ∼25 nm steps was observed. Interestingly, a small population (*∼*4%) of ∼21 nm events was also present in the high-force pulse, corresponding to the scission of a non-native disulfide bond formed between two contiguous domains within the polyprotein chain^40^ (Supplementary Fig. 1). As a control experiment, we verified that the oxidized protein was able to independently refold under high-pH conditions, as shown in Supplementary Fig. 2. Altogether, our experiments qualitatively show that an individual disulfide bond is able to reform in the absence of catalytic enzymes.

We next examined whether such oxidative folding scenario could also be reproduced when the specific location of the disulfide bond within the structure of the I27 protein was changed. We repeated the experiments reported in Fig. 1 on a distinct polyprotein construct, (I27_G32C–A75C_)_8_, in which the cysteine mutations were engineered in different positions of the I27 sequence. The resulting trajectories (Supplementary Fig. 3) qualitatively certified the successful reformation of the disulfide bond along the folding pathway.

### The kinetics of disulfide bond reformation

**Figure 2:**
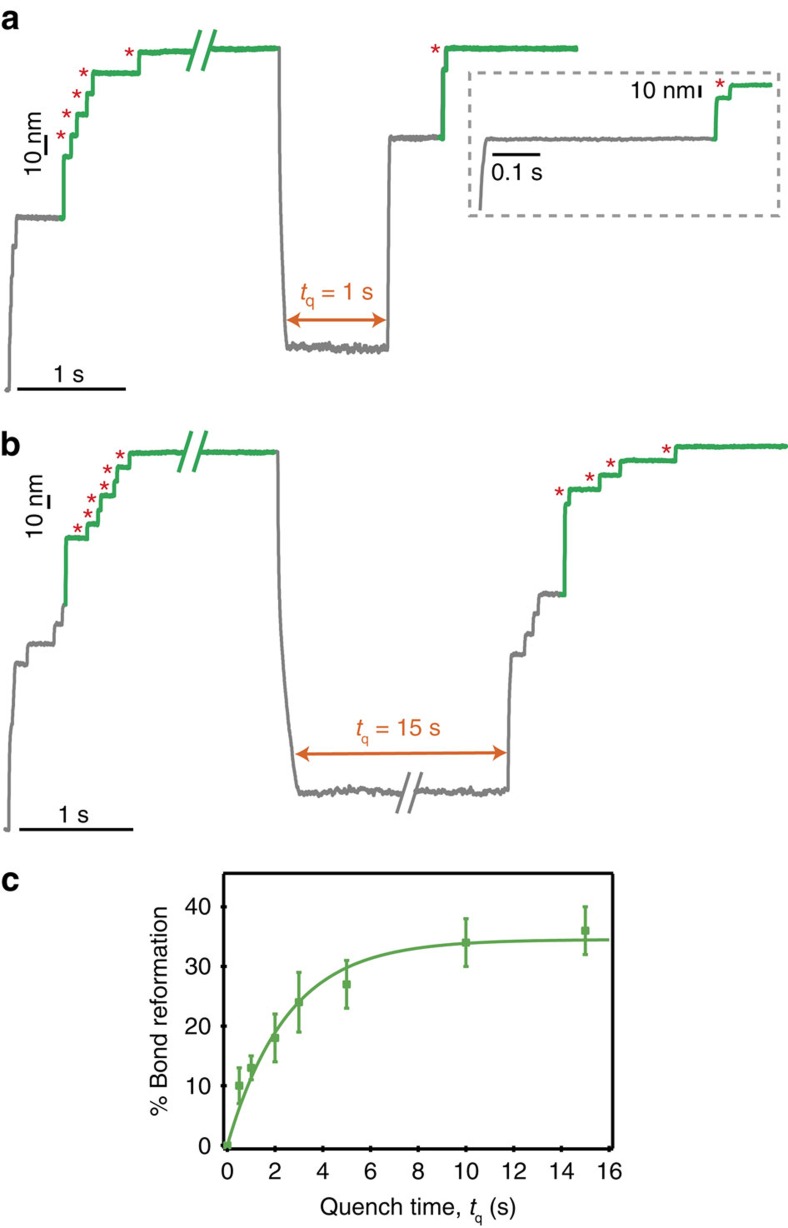
Kinetics of disulfide bond reformation. (**a**,**b**) The percentage of disulfide reformation, fingerprinted by the presence of ∼10 nm steps (red asterisks), increases with *t*_q_. **a** illustrates a particular trajectory showing that the reformation of the disulfide bond (marked by the 10 nm step) occurred in the absence of protein refolding, since the 10 nm step, corresponding to the rupture of the disulfide bond, is not preceded by a 15 nm step, hallmark of protein refolding. This suggests that, in this particular trajectory, we interrupted the oxidative folding process at an instant whereby the reformation of the disulfide bond had occurred but the protein did not have yet time to fold. (**c**) The percentage of disulfide bond reformation increases exponentially with *t*_q_ with an associated rate constant of *k*_ref_=0.45 s^−1^ (solid green line, *χ*^2^_red_=0.513).

To quantify the kinetics of disulfide bond reformation, we repeated the experiments shown in Fig. 1 and varied the quench time (*t*_q_) within the range spanning *t*_q_=0.5–15 s (Fig. 2). As observed in the individual trajectories shown in Fig. 2a,b, the longer the protein was allowed to refold in the absence of force, the higher the percentage of disulfide bonds that reformed (hallmarked by the appearance of ∼10 nm steps in the test pulse). As shown in Fig. 2c, the per cent of disulfide bond reformation increased exponentially with the quench duration, suggesting that the reformation of the protein disulfide bond is a two-state process with an associated characteristic time of *k*_ref_=0.45 s^−1^. This is consistent with a scenario where the nucleophilic thiol would attack the electrophilic -SOH centre via a concerted S_N_2 reaction, occurring through a well-defined transition state^41^,^42^, to reform the disulfide bond. Noteworthy, the measured reformation rate is expected to be pH dependent, since the nucleophilic capacity of the free thiolate to attack the electrophilic -SOH will be largely dependent on the thiolate being completely deprotonated. However, given the expected p*K*_a_∼8 for each free cysteine, we do not anticipate these rates to be significantly affected in our high-pH experiments, although this might have an effect at lower pH values. Remarkably, the measured rate of disulfide reformation for the (I27_G32C–A75C_)_8_ construct (*k*_ref_=0.42 s^−1^) was found to be almost identical to that measured for the (I27_E24C–K55C_)_8_ polyprotein, thus demonstrating that the disulfide bond reformation kinetics is not significantly altered by changing the position of the disulfide bond within the protein structure (Supplementary Fig. 3).

### Reactivity of -SOH depends on solvent accessibility

**Figure 3:**
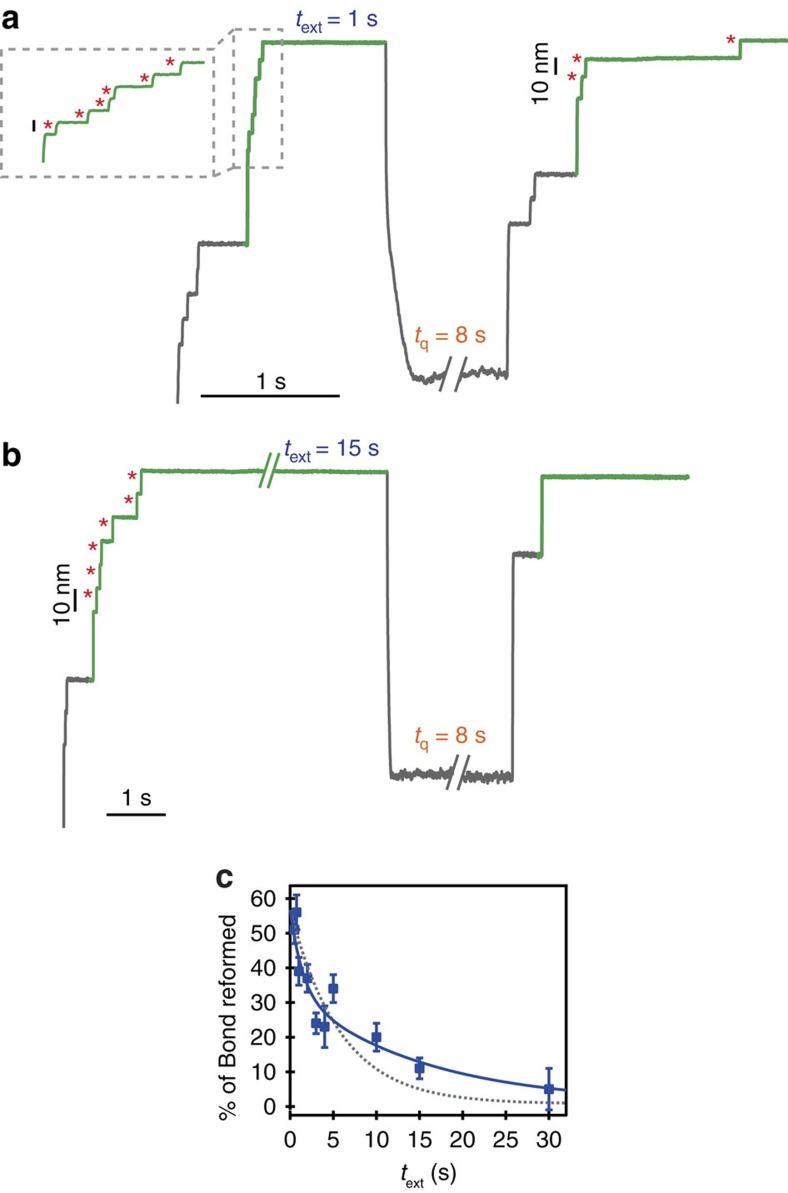
*S*-sulfenylation of cryptic cysteines inhibits oxidative folding. (**a**,**b**) Increasing the time, *t*_ext_, the protein is reduced and stretched at high force (*F*=500 pN) results in a drastic decrease of successful oxidative folding (marked by the decrease of both ∼10 nm steps, red asterisks, and ∼15 nm steps in the test pulse). (**c**) While the decrease in the % of disulfide bond reformation as a function of *t*_ext_ can be fitted with a single exponential (dotted grey line) with a rate constant *k*=0.17 s^−1^ (*χ*^2^_red_=5.02), the trend is best described (*χ*^2^_red_=2.78) by a double exponential fit (solid blue line), with associated rate constants *k*_1_=0.065 s^−1^ and *k*_2_=0.695 s^−1^, reminiscent of two competing processes occurring concomitantly.

While generally short, the life-time of -SOH has been described to crucially depend on the degree of solvent exposure^20^,^43^. Force-clamp AFM allows to explore new protein conformations that are not usually sampled in classical ensemble experiments conducted in the bulk^44^. In particular, the force-triggered low-entropy, highly extended unfolded state fully exposes the backbone residues to the solvent environment^45^. To interrogate the conformation-dependent reactivity of -SOH, we systematically changed the time the fully reduced and mechanically stretched protein was exposed to the solvent, *t*_ext_, thereby increasing the probability to be chemically modified, while keeping the quench time constant (*t*_q_=8 s, Fig. 3a,b). Leaving the protein stretched for *t*_ext_=0.5 s resulted in a ∼55% disulfide bond reformation. Substantially increasing the extended time up to *t*_ext_=30 s considerably decreased the reformation probability down to ∼5%. The absence of disulfide bond reformation inevitably resulted in protein misfolding, since we never observed the presence of a 15 nm step without the companion 10 nm step in the test pulse. The evolution of the per cent of disulfide bond reformation as a function of *t*_ext_ showed a marked, fast decrease (Fig.3c). While fitting a single exponential to the data qualitatively described the time-course of the process with an associated decaying rate of *k*=0.17 s^−1^ (*χ*^2^_red_=5.02), a double exponential (*k*_1_=0.065 s^−1^ and *k*_2_=0.695 s^−1^) slightly increased the goodness of the fit (*χ*^2^_red_=2.78, Supplementary Fig. 4), reminiscent of two independent reaction pathways occurring concomitantly.

**Figure 4:**
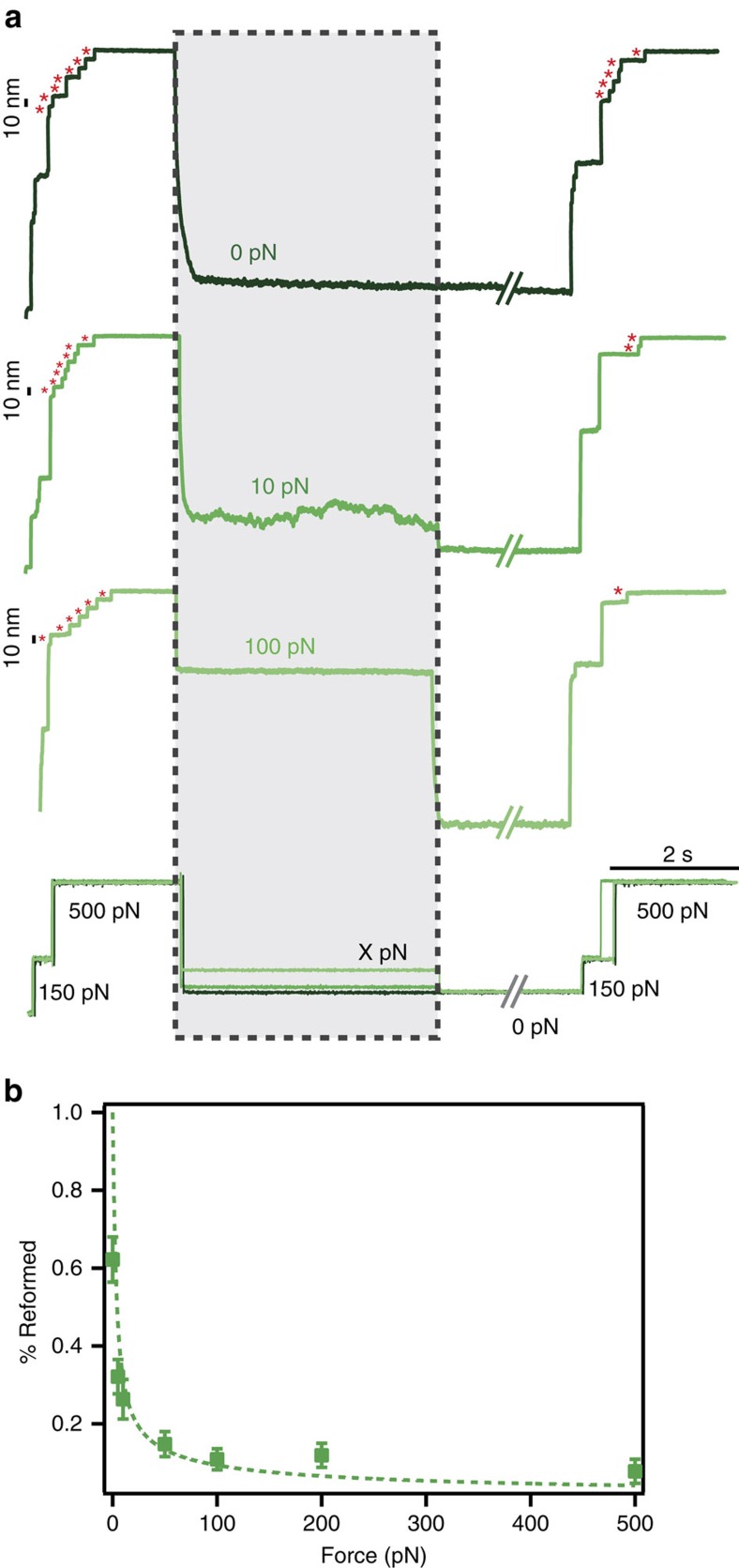
The chemical evolution of sulfenic acid is force-dependent. The degree of solvent exposure is tuned by the pulling force, which determines the (I27_E24C–K55C_)_8_ protein end-to-end length. (**a**) A six-pulse force protocol probes the effect of force and solvent exposure on the outcome of oxidative folding. The number of 10 nm steps (red asterisks) significantly decreases with increasing the force applied to the protein after the sulfenic acid is formed (grey box), varying from 0 pN (dark green) to 100 pN (light green). (**b**) The percentage of disulfide bond reformation shows a drastic decrease with an increase in the stretching force, and the evolution can be captured by the worm-like chain (WLC) model of polymer elasticity (*P*=1.2 nm). This trend is indicative of the distinct reactivity of sulfenic acid as a function of its exposure to the solvent. At low forces the local protein environment shields the sulfenic acid, allowing it to condense into a reformed disulfide bond that ensures successful oxidative folding. By contrast, at higher forces the sulfenic acid is fully exposed to the solution and undergoes further chemical modification, ultimately leading to protein misfolding, hallmarked by the inability of the protein to recover its native mechanical stability.

To directly quantify the relationship between the degree of solvent exposure and -SOH reactivity, we systematically varied the stretching force after triggering the formation of -SOH. Using a six-pulse force protocol (Fig. 4), the initial two pulses unfolded the protein (150 pN, 0.3 s) and triggered the rupture of the disulfide bond within a short time (500 pN, 2 s). The force was then varied within a range spanning 0–500 pN during 4 s (grey box) before being withdrawn (0 pN, *t*_q_=8 s) to trigger collapse^39^ and oxidative folding. As before, the folding success was probed by two high force test pulses (150 pN followed by 500 pN). The folding trajectories shown in Fig. 4a qualitatively show that the number of 10 nm steps observed in the test pulse (red asterisks) significantly decreased as the stretching force was increased. This tendency was quantitatively verified in Fig. 4b, displaying the percentage of disulfide bond reformation as a function of the stretching force. Crucially, the observed trend can be nicely reproduced by the worm-like chain (WLC) model of polymer elasticity (using a persistence length *P*=1.2 nm)^45^, which relates the protein's end-to-end length with the pulling force. At each defined force, the WLC model defines an equilibrium end-to-end conformation that is characterized by a different degree of solvent exposure of the protein residues^46^. Altogether, these experiments demonstrate that the reactivity of -SOH—intimately related to the oxidative folding fate of the protein— is largely determined by the protein's conformation.

To investigate the molecular origin of the chemical modification that prevented the reformation of the disulfide bond at high pulling forces, we conducted MS measurements on the monomeric I27_E24C–K55C_ protein under high-pH conditions. MS spectra revealed the presence of both sulfinic and sulfonic acid, thus suggesting the successive irreversible oxidation of -SOH (Supplementary Figs 5 and 6 and Supplementary Table 1). Notably, the presence of aldehyde (with a concomitant loss of H_2_S) was also detected with good evidence (Supplementary Fig. 7 and Supplementary Table 1). Indeed, aldehyde formation has been long reported as a sub-product of the decomposition of -SOH under high alkali conditions^28^,^47^,^48^. While both the protein conformation and the timescales sampled in the MS and single-molecule mechanics experimental approaches are certainly different, the MS data provide excellent complementary information that illustrates the most plausible chemical processes responsible for the misfolding events captured in the nanomechanical experiments, greatly affecting the folding and elastic properties of the individual titin polyproteins.

### Dimedone binding confirms the presence of -SOH

**Figure 5:**
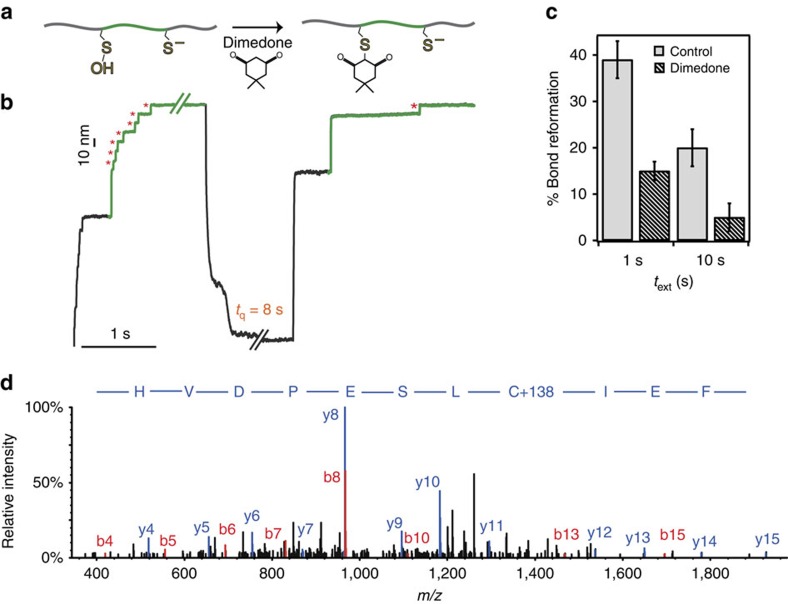
Dimedone selectively targets sulfenic acid and blocks oxidative folding. (**a**) The newly formed sulfenic acid moiety can be selectively trapped by dimedone. (**b**) When an individual (I27_E24C–K55C_)_8_ polyprotein is reduced and stretched in the presence of a 30 mM solution of dimedone, the subsequent test pulse shows a dramatic decrease in the presence of both ∼10 nm (red asterisks) and ∼15 nm steps, implying that oxidative folding is hindered. (**c**) The percentage of disulfide bond reformation (and hence of successful folding) decreases with *t*_ext_, the time the protein remains extended and stretched at a high force. For *t*_ext_=1 s, the % reformation decreases down to 15.6±2.3% in the presence of dimedone. On increasing *t*_ext_=10 s, the percentage of reformation is further reduced (6.0±3.3%). (**d**) The MS/MS fragmentation spectra of an I27_E24C–K55C_ monomer under high-pH conditions provided unambiguous evidence of the presence of the dimedone, hallmarked by a cysteine mass shift of Δ*m*/*z*=+138 Da.

To provide a final, unambiguous proof that -SOH is actively involved in the molecular mechanism driving the reformation of disulfide bonds, we directly tested the effect of dimedone on the outcome of our oxidative folding assay. Dimedone has been vastly demonstrated to effectively react with -SOH (but not with thiols) with high specificity (Fig. 5a)^7^,^15^. Addition of 30 mM of dimedone to the measuring solution markedly blocked both the reformation of the disulfide bond and the folding of the protein (Fig.5b), decreasing the bond reformation efficiency down to ∼15% (Fig. 5c). This drastic decrease in the reformation (and subsequent refolding) efficiency was further exacerbated (down to a residual ∼6%) when the protein was left fully extended at high forces for a longer period of time, *t*_ext_=10 s, promoting dimedone binding (Fig. 5c). By contrast, dimedone did not prevent the protein from successfully refolding when the disulfide bond remained oxidized (Supplementary Fig. 8). To indisputably verify the presence of the covalent S-dimedone adduct, we conducted MS measurements on the monomeric I27_E24C–K55C_ protein under high-pH conditions. The resulting MS/MS fragmentation spectra identified, for both 24 and 55 cysteines, a shift in Δ*m*/*z*=+138 Da (Fig. 5d, Supplementary Fig. 9 and Supplementary Table 2), which confirmed the presence of dimedone.

## Discussion

Oxidative stress defines elevated intracellular levels of reactive oxygen species (ROS)—mostly including superoxide anion, hydrogen peroxide and hydroxyl radicals—that cause damage to DNA, lipids and proteins^49^. While ROS were originally known for oxidizing various cellular compartments and promoting aging and a broad range of pathologies, more recent evidence shows that ROS also acts as signalling molecules that regulate basic cellular processes including growth, differentiation and cell migration^26^,^50^. It is increasingly clear that a major mechanism of redox signalling is the dynamic regulation of protein function by the chemiselective oxidation of cysteine residues. The first product of oxidation is Cys-SOH, exhibiting an oxidation state of (0). Despite its ephemeral nature, -SOH has been trapped in a number of crystallized proteins^12^,^51^. These carry a wide range of functionalities^12^, encompassing enzyme catalysis (such as protein tyrosine phosphatases^52^, kinases^53^ and cysteine proteases^54^), transcription factors (including OxyR^55^, Yap1 (ref. 56) and NF-kB^57^) and ion channels (such as ORAI1 (ref. 58) and Kv1.5 (ref. 59)). This progress notwithstanding these structures provide a static snapshot, and the fast dynamics of such important post-translational modification often evaded characterization.

The relatively small number of crystallized proteins contrasts with the increasingly larger number of proteins where -SOH has been identified *in vivo*. Indeed, the dimedone-based cell-permeable chemical probe DAz-2, capable of detecting SOH-modified proteins directly in living cells^50^, has allowed for a comprehensive analysis of the cellular ‘sulfenome'^18^, revealing the presence of (>1,000) S-sulfenylation sites on more than 700 proteins in intact cells, highly prevalent in different cancer cell lines^14^,^60^. Importantly, these newly identified protein targets largely expand the diversity of functionalities of the already identified sulfenylated proteins. Therefore, unveiling the molecular mechanisms defining -SOH reactivity is an essential requirement for the understanding of redox regulation in cells.

Our single-molecule approach allows independent measurement of the protein folding and disulfide bond formation processes, enabling us to directly visualize the molecular events defining the complex kinetics of the oxidative folding mechanism. The crucial role of disulfide reformation in protein refolding was further probed in experiments on the pre-reduced (I27_E24C–K55C_)_8_ polyprotein at pH=12.8. While the oxidized protein was able to refold under high-pH conditions (Supplementary Fig. 10), the folding efficiency of the pre-reduced protein was significantly decreased down to ∼20%, exhibiting a seemingly slow folding kinetics, *k*_fold_=0.10 s^−1^ (Supplementary Fig. 10). This result contrasts with the much higher folding efficiency of the sulfenylated protein, which folds on the reformation of the disulfide bond (*k*_ref_=0.45 s^−1^), ultimately setting the associated folding rate (*k*_fold_=0.46 s^−1^). These experiments demonstrate that while the protein is mostly unable to successfully refold on its own under high pH, the reformation of the disulfide bond dramatically increases its folding efficiency. In this sense, -SOH emerges as a key molecular player able to rescue the protein from misfolding by forming a protective disulfide bond that allows the protein to successfully refold. This kinetic scheme seems to differ from the enzyme-mediated oxidative folding findings catalysed by PDI^40^ and DsbA^61^, whereby the same I27 protein mutant was able to refold on its own while keeping the catalytic enzyme attached through a mixed disulfide conformation before the disulfide bond was successfully reformed at a later stage, the overall kinetics being largely controlled by the off-rate dynamics of the catalytic enzyme.

We postulate that the oxidative folding mechanism promoted by -SOH condensation with a neighbouring intramolecular thiolate is mechanistically different from the process where the reformation of the disulfide bond occurs through a mixed disulfide intermediate, which characterizes the enzyme-catalysed oxidative folding process^22^. To test this hypothesis, we repeated the force-quench experiments at pH=7.2 using the small thiol 3-mercapto-1-propanol as the attacking nucleophile^41^ (Supplementary Fig. 11). In this case, the rupture of each individual disulfide bond resulted in the formation of a thiolate and the newly formed mixed disulfide. Unlike the case where -SOH could readily reform the cleaved disulfide, thus triggering the protein to correctly fold, when 3-mercapto-1-propanol was used as a nucleophile, the number of reformation events was vanishingly small. By contrast, these experiments highlighted (albeit with a relatively low abundance) the presence of 25 nm steps in the test pulse. Altogether, our experiments demonstrate the unique capabilities of -SOH to form a disulfide bond employing a chemical mechanism that is fundamentally different from that occurring during enzyme-mediated protein folding^22^,^40^. In this novel -SOH-mediated scenario, the newly formed disulfide reduces the conformational space of the protein, and subsequently triggers almost ‘instantaneous' folding of the remaining protein in the timescale of our experiments. Thus, the kinetics of disulfide bond formation is the rate-limiting step that defines the overall kinetics of the non-enzymatic oxidative folding process. It is certainly remarkable that enzyme-free oxidative folding occurs under high pH conditions at a similar rate (*k*_ref_=0.45 s^−1^) than that catalysed by a dedicated enzyme such as PDI (*k*_ref_=0.35 s^−1^) and DsbA (*k*_ref_=0.70 s^−1^)^40^,^61^.

Our experiments demonstrate that the probability of reforming the disulfide bond increases exponentially with time (Fig. 2). This implies that, when the protein lies within the non-folded collapsed conformation after the force-quench, the highly reactive -SOH moiety is sterically stabilized within the microenvironment of the collapsed protein, most likely through becoming cryptic to the solvent. This situation contrasts with the fast evolution of the -SOH in the fully extended state (Fig. 3). At the present stage it is difficult to unambiguously pinpoint the exact origin of the fast reactivity undergone by the solvent-exposed -SOH. Due to the relatively short timescale of our single-molecule experiments, it is unlikely that the progression to higher oxidation state species is favoured, and hence we hypothesize that the aldehyde formation mechanism might be prevalent^28^,^47^,^48^, which would account for the main pathway described by the single exponential fit. Given the slightly higher accuracy of the double exponential fit, we postulate a possible competition between the high oxidation pathway (promoted by the dissolved oxygen in solution) and the aldehyde formation mechanism, exhibiting similar rate constants. Noteworthy, we cannot rule out the possibility that a fraction of the aldehyde population found in our MS experiments is a result of the direct alkali-mediated cleavage of the disulfide bond through an α-elimination mechanism, which competes with a β-elimination and the hydroxyl-mediated S_N_2 nucleophilic attack^27^,^28^,^29^,^62^ (Supplementary Fig. 12). Indeed, our MS spectra show evidence of the three competing pathways (Supplementary Figs 7, 13 and 14 and Supplementary Table 1). However, it is very likely that, in the presence of force, the hydrolysis (S_N_2) pathway is largely favoured over the elimination route, as suggested by recent quantum mechanical simulations under force^63^, in which case the presence of aldehyde is likely to stem mostly from the evolution of -SOH. Further verification of the plausible competing reaction pathways under force, probably guided by *ab initio* simulations, might provide a comprehensive understanding of this complex reactivity landscape. In any case, while the high-pH conditions tested in our experiments probably trigger a faster -SOH reactivity towards the aldehyde product than one would perhaps expect under more physiologically relevant conditions, our experiments readily demonstrate that the reactivity of -SOH is largely conformation dependent, crucially contributing to the folding fate of the protein. We speculate that this mechanism might hold true in situations where -SOH is triggered by other biologically occurring oxidative species such as H_2_O_2_. To test this hypothesis, we conducted folding experiments on the wt-I27 immunoglobulin module (containing two cryptic and native cysteines in positions 47 and 63 that do not form a disulfide bond) in the presence of 300 μM H_2_O_2_ at neutral pH (Supplementary Fig. 15). Stretching and unfolding the (wt-I27)_8_ polyprotein exposed the cryptic cysteines to the oxidative conditions. Quenching the force and re-unfolding back again resulted in (i) the misfolding of the protein, fingerprinted by the absence of recovery of mechanical stability, and (ii) the inability of the protein to completely re-extend up to its unfolded contour length, suggestive of the presence of a stiff, non-native (probably interdomain) disulfide bond. Controls were performed with the double mutant (I27_C47A–C63A_)_8_ polyprotein (devoid of both cysteines) showing full refolding and complete protein extensibility. These experiments, conducted in the presence of the less controllable yet physiologically more relevant H_2_O_2_ oxidative agent, strongly suggest that, when solvent exposed on mechanical unfolding^64^, cryptic cysteines undergo post-translational modifications that markedly block protein folding, while lending further support to the SOH-mediated mechanism of disulfide bond formation that we investigated using the high-pH experimental strategy.

**Figure 6:**
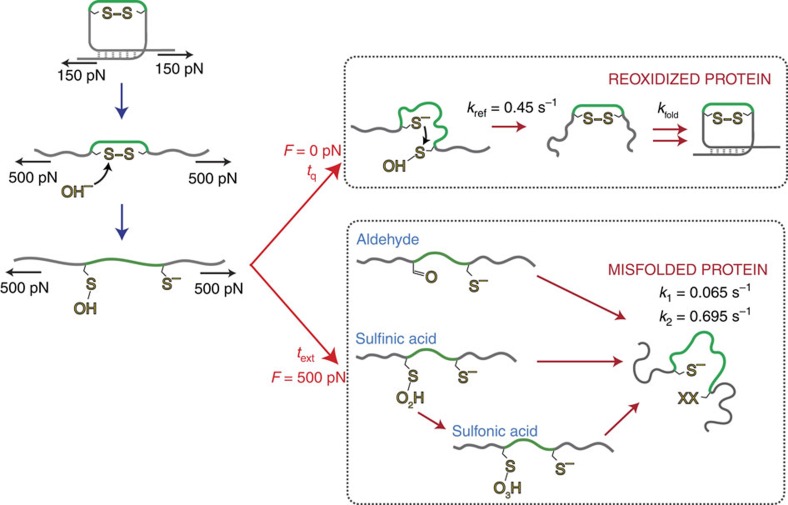
Kinetic scheme for the force-triggered, conformation-dependent reactivity of sulfenic acid and its implications for oxidative protein folding. On the application of mechanical force, the hydroxide anions rupture a protein disulfide bond, creating a sulfenic acid and a thiolate. In the absence of force (*F*=0 pN) during the quench time (*t*_q_) the sulfenic acid moiety remains cryptic in the protein's collapsed state. Under these conditions, the longer *t*_q_, the higher the probability the thiolate anion can attack the sulfenic acid moiety (in the absence of dimedone), thus creating a disulfide bond with an associated rate of *k*_ref_=0.45 s^−1^. The reformation of the disulfide bond triggers the ‘instantaneous' refolding of the protein (essentially set by the disulfide bond reformation rate). By contrast, when the protein is reduced and stretched at high force (*F*=500 pN), sulfenic acid is fully exposed to the solvent and undergoes further modification either to the formation of sulfinic acid (which may be further oxidized to sulfinic acid) or to aldehyde. Such further reactivity of sulfenic acid results in irreversible misfolding of the reduced (I27_E24C–K55C_)_8_ polyprotein, with a consequent decrease in its stiffness properties.

Taken together, our nanomechanical experiments put forward a rather unique kinetic scheme (Fig. 6) that captures the intricate conformation-dependent reactivity of the transitory -SOH intermediate, directly linked with the folding fate of the protein. In the case of titin, a protein that is physiologically exposed to mechanical force, such SOH-regulated folding scenario might have important consequences for the functional protein elasticity.

Titin is a molecular ruler that determines the passive elasticity of muscle, and is key to ensure, for example, cardiac function^65^. Indeed, alterations in the stiffness of cardiac titin are related to a number of myopathies, such as diastolic dysfunction^66^. The I-band region of titin is formed by a compliant, unstructured region (PEVK and N2B) intercalated between folded immunoglobulin domains, which are mechanically resistant^67^. To adapt to the mechanical perturbations induced by the heartbeats, titin needs to reversibly modulate its length by entropic extension/recoil of the compliant region and unfolding/refolding (at least of the mechanically weak) immunoglobulin domains^64^. On immunoglobulin domain unfolding, the stiffness of the protein is greatly reduced, and it is increased back again on refolding. Recent pioneering evidence shows that S-glutathionylation of cryptic cysteines blocks titin refolding^64^. Our results are likely to add S-sulfenylation to the toolbox of post-translational modifications that regulate titin elasticity; whereas cysteine hyperoxidation irreversibly suppresses Ig27 refolding, thus severely decreasing its stiffness, the reformation of a protective disulfide bond has the opposing effect. In this sense, -SOH would act as ‘mechanical switch', helping titin adapt its elasticity in response to the fluctuating oxidative stress conditions. We hence speculate that -SOH might work as an active redox sensor that directly regulates protein elasticity.

## Methods

### Protein engineering

The (I27_E24C–K55C_)_8_, (I27_G32C–A75C_)_8_, (I27)_8_ and (I27_C47A–C63A_)_8_ polyproteins were subcloned using the *Bam*HI, *Bgl*II and *Kpn*I restriction sites. The multidomain proteins and the I27_E24C–K55C_ monomer were cloned into the pQE80L (Qiagen) expression vector, and transformed into the BLR(DE3) *Escherichia coli* expression strain. The cells were grown in LB broth supplemented with 100 μg ml^−1^ ampicillin at 37 °C. After reaching an OD_600_ of ∼0.6, the cultures were induced with Isopropyl β-D-1-thiogalactopyranoside (1 mM) and incubated overnight at 20 °C. After collecting the cells, disruption using a French Press was performed. The polyproteins from the lysate were purified by metal affinity chromatography on Talon resin (Clontech) followed by gel filtration using a Superdex 200 10/300 GL column (GE Biosciences). Protein concentration in the samples was estimated using the Bradford protein assay.

### Force spectroscopy

Single-molecule force-clamp spectroscopy AFM experiments were conducted at room temperature using both a home-made set-up^68^ and a commercial Luigs and Neumann force spectrometer^69^. The sample was prepared by depositing 1–10 μl of protein in PBS solution (at a concentration of 1–10 mg ml^−1^) onto a freshly evaporated gold coverslide. Each cantilever (Si_3_N_4_ Bruker MLCT-AUHW) was individually calibrated using the equipartition theorem, giving rise to a typical spring constant of ∼12–17 pN nm^−1^. Single proteins were picked up from the surface and pulled at a constant force. Individual polyproteins were fished by pushing the cantilever onto the surface exerting a contact force of 500–1,500 pN so as to promote the non-specific adhesion of the proteins on the cantilever surface. The piezoelectric actuator was then retracted to produce a set deflection (force), which was set constant throughout the experiment (∼8–45 s) thanks to an external, active feedback mechanism while the extension was recorded. The force feedback was based on a proportional, integral and differential amplifier whose output was fed to the piezoelectric positioner. The feedback response is limited to ∼3–5 ms. Thanks to the high-resolution piezoelectric actuator, our measurements of protein length have a peak-to-peak resolution of ∼0.5 nm. Data of the force traces was filtered using a pole Bessel filter at 1 kHz. In all instances the protein was unfolded at 150 pN for 0.5 s, followed by a high-force pulse of 500 pN, except when the protein was extended for very short times (0.5 and 0.75 s) when a pulse of 800 pN was applied. Experiments were carried out in a sodium phosphate buffer solution, specifically, 50 mM sodium phosphate (Na_2_HPO_4_ and NaH_2_PO_4_), 150 mM NaCl. The pH was adjusted at pH=7.4 and pH=12.8. The final pH value in each solution was adjusted by adding the required amounts of NaOH solutions. Measuring solutions were filtered through a 0.2-μm membrane before each experiment. In the case of the dimedone experiments, 5,5-Dimethyl-1,3-cyclohexanedione (Sigma-Aldrich, 95%) was dissolved in pH=12.8 PBS buffer to give a final concentration of 30 mM. The pH of the dimedone solution was checked before each experiment. 3-Mercapto-1-propanol (Sigma-Aldrich, 95%) was freshly prepared before every experiment, dissolved in pH=7.4 PBS buffer to give a final concentration of 400 mM. Pre-reduced (I27_E24C–K55C_)_8_ polyproteins were obtained by incubating the polyproteins in 10 mM Tris(2-carboxyethyl)phosphine hydrochloride (TCEP, Sigma-Aldrich) for 20 min before AFM experiments. Hydrogen peroxide solutions (H_2_O_2_, Sigma-Aldrich) were obtained by dissolving the 30% (v/v) solution in PBS to a final concentration of 300 μM H_2_O_2._

### Data analysis

All data were recorded and analysed using custom software written in Igor Pro 6.0 (Wavemetrics). For all polyproteins, only recordings showing the signature of at least 5 unfolding events (15 nm steps) followed by 5 reduction events (10 nm steps) were analysed. We considered only recordings corresponding to the test pulse in which the protein was extended back to the same full unfolded length characterizing the initial pulse, to ensure that the same protein was completely stretched in the two pulses. No traces that included unfolding events during the high force pulse were included in the analysis. The number of independent observations, *N*, for a particular condition was counted as the total number of protein domains (as observed in the initial pulse). S.e.m. for the refolding fraction was estimated through the boostrap method, where each recording was treated as an independent data point. s.e.m. for the fit parameters was determined as standard error for the coefficient in the fit, accounting for the measurement errors of the individual data points. To assess the goodness of the fits in Fig. 2 and Fig. 3, we conducted reduced *χ*^2^, *χ*^2^_red_, analysis. Data in Fig. 4b was compared with the WLC model of polymer elasticity^70^, using as fixed parameters *L*_c_=28.5 nm, *P*=1.2 nm.

### MS experiments

*Enzymatic digestion*. The monomeric protein samples were prepared by diluting 20 μl of protein (0.5 μg μl^−1^) in 20 μl of either PBS pH 7.4, PBS pH12.8 or PBS pH12.8+30 mM dimedone. The high-pH samples had a calculated final pH=12.5. In-solution digestion with trypsin of 5 μg of total protein monomers was performed before subsequent analysis by MS. Trypsin digestion at a ratio of 1:20 (enzyme:substrate) was carried out for 2 h at 37 °C.

*LC-MS/MS*. Chromatographic separations were performed using an EASY NanoLC system (ThermoFisherScientific, UK). Peptides from a total protein amount of 2 μg on column were resolved by reversed phase chromatography on a 75 μm C18 column using a three step linear gradient of acetonitrile in 0.1% formic acid. The gradient was delivered to elute the peptides at a flow rate of 300 nl min^−1^ over 60 min. The eluate was ionized by electrospray ionization using an Orbitrap Velos Pro (ThermoFisherScientific, UK) operating under Xcalibur v2.2. The instrument was programmed to acquire in automated data-dependent switching mode, selecting precursor ions based on their intensity for sequencing by collision-induced fragmentation using a Top20 CID method. The MS/MS analyses were conducted using collision energy profiles that were chosen based on the mass-to-charge ratio (*m/z*) and the charge state of the peptide.

### MS database searching

Raw MS data were processed into peak list files using Proteome Discoverer (ThermoScientific; v1.4). Processed raw data was searched using the Mascot search algorithm (www.matrixscience.com) against an in-house database containing the sequence of the mutant protein monomer. The data was also searched using the Error Tolerant function which by iterating through a comprehensive list of chemical and post-translational modifications, determines the mass of additions or losses from the residues in the peptide sequences without prior knowledge of the expected modifications. LC/MS/MS analysis has successfully identified 100% sequence coverage of the mutant protein monomer with various forms of oxoacid modifications. The Mascot database searching algorithm applies a 95% probability CI in the MOWSE scoring that is an identification threshold. Mascot search output files were uploaded into the Scaffold spectral visualization software (v.4.4.6; www.proteomesoftware.com) which allows statistical filtering of the data at the protein and peptide level for which these filters were applied to the data at a the lowest stringency of 5% confidence interval (CI) for minimum protein, 0% CI for peptide values and minimum one peptide assignment. This was chosen to include all assigned peptides that may have fallen below the database applied 95% CI due to poor fragmentation matching and incorrect sequence assignment. All fragmentation spectra were verified manually to determine correct assignment of sequences and post-translation modifications.

Initial data searching concentrated on finding -SOH modifications after the addition of the cyclic diketone compound, dimedone. Dimedone specifically traps this oxoacid form and binds covalently to the cysteine residue. This binding with the subsequent loss of two protons adds a mass of 138.1 Da to the residue, which is detected on the peptide by fragmentation in MS/MS mode in the mass spectrometer.

Under conditions of high pH and without the presence of dimedone to specifically trap the -SOH, there is possible evolution of the oxoacids to be present in their different forms. The protein monomer treated with high pH alone was searched against the in-house database, which included the modifications -SOH, sulfinic acid and sulfonic acid. Further in-depth searching of the database was also performed with the modifications aldehyde, dehydroalanine and sulfide. Database searching assigned 99% sequence coverage of the protein monomers and also assigned various oxoacid modifications on the cysteine residues of interest.

Further analysis was performed using a fresh sample of monomers in a high pH only environment and treated with enzymatic digestion as previously described. This sample was digested almost immediately following the addition of the high-pH buffer. This was performed to determine whether the previously identified oxoacid modifications were present at point of treatment, or if they had evolved over the length of time it had spent in the buffer before trypsin digestion. Almost complete sequence coverage was achieved once again following database searching of the mass spectrometer raw data. Oxoacid cysteine modifications sulfonic and sulfinic acid were identified at residue Cys55 as were the products of both α-elimination, aldehyde and β-elimination, persulfide and dehydroalanine.

### Data availability

The MS proteomics data have been deposited to the ProteomeXchange Consortium via the PRIDE partner repository with the dataset identifier PXD004514. Additional information and the data supporting this research, including the single-molecule nanomechanics experiments, can be obtained from the corresponding author on request.

## Additional information

**How to cite this article:** Beedle, A.E.M. *et al*. Protein S-sulfenylation is a fleeting molecular switch that regulates non-enzymatic oxidative folding. *Nat. Commun.* 7:12490 doi: 10.1038/ncomms12490 (2016).

## Supplementary Material

Supplementary Information Supplementary Figures 1-15 and Supplementary Tables 1-2
