# Supplementary material for: Protein S-sulfenylation is a fleeting molecular switch that regulates non-enzymatic oxidative folding
Source: Nat Commun. 2016 Aug 22;7:12490. doi: 10.1038/ncomms12490 (PMC4996944; doi:10.1038/ncomms12490)
Supplement: Supplementary Information — Supplementary Figures 1-15 and Supplementary Tables 1-2 [file ncomms12490-s1.pdf]

## Supplementary Figures

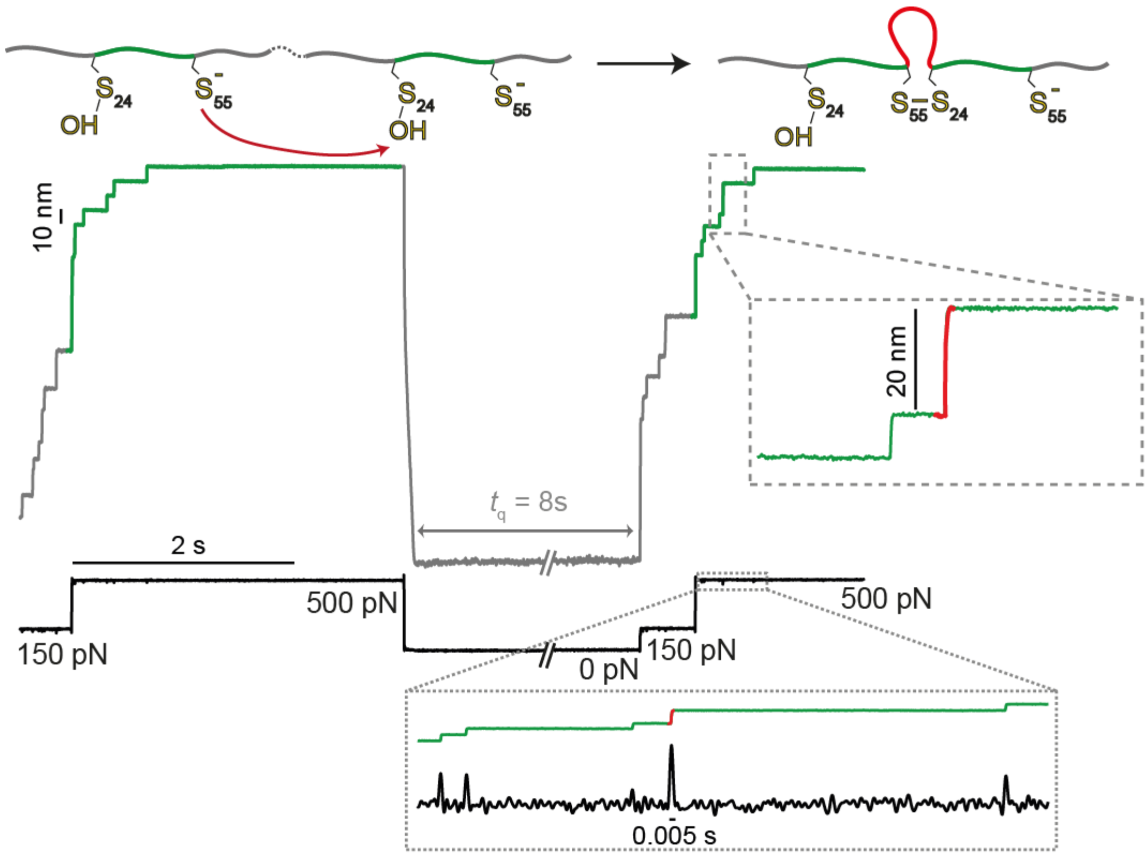


**Supplementary Figure 1. Capturing misfolding events triggered by the formation of non-native disulfide bonds between contiguous Ig domains within the polyprotein chain.** The low-probability events (~4%) observed in the step size histogram corresponding to the *test* pulse (Fig. 1e) can be explained by the formation of a disulfide bond between two cysteines belonging to two independent, neighbouring domains of the $(I27_{E24C\text{-}K55C})_8$ polyprotein. The individual oxidative folding trajectory fingerprints the presence of such new, ill-formed disulfide bond by the presence of a 20 nm step (red) in the *test* pulse occurring at 500 pN. This step size corresponds to the extension of the 58 amino acids trapped behind a disulfide bond formed between cysteine 55 and cysteine 24 of two neighbouring Ig domains. *Inset:* Magnification of the force channel demonstrating that the feedback response time of the instrument (upward spikes) is less than 5 ms.

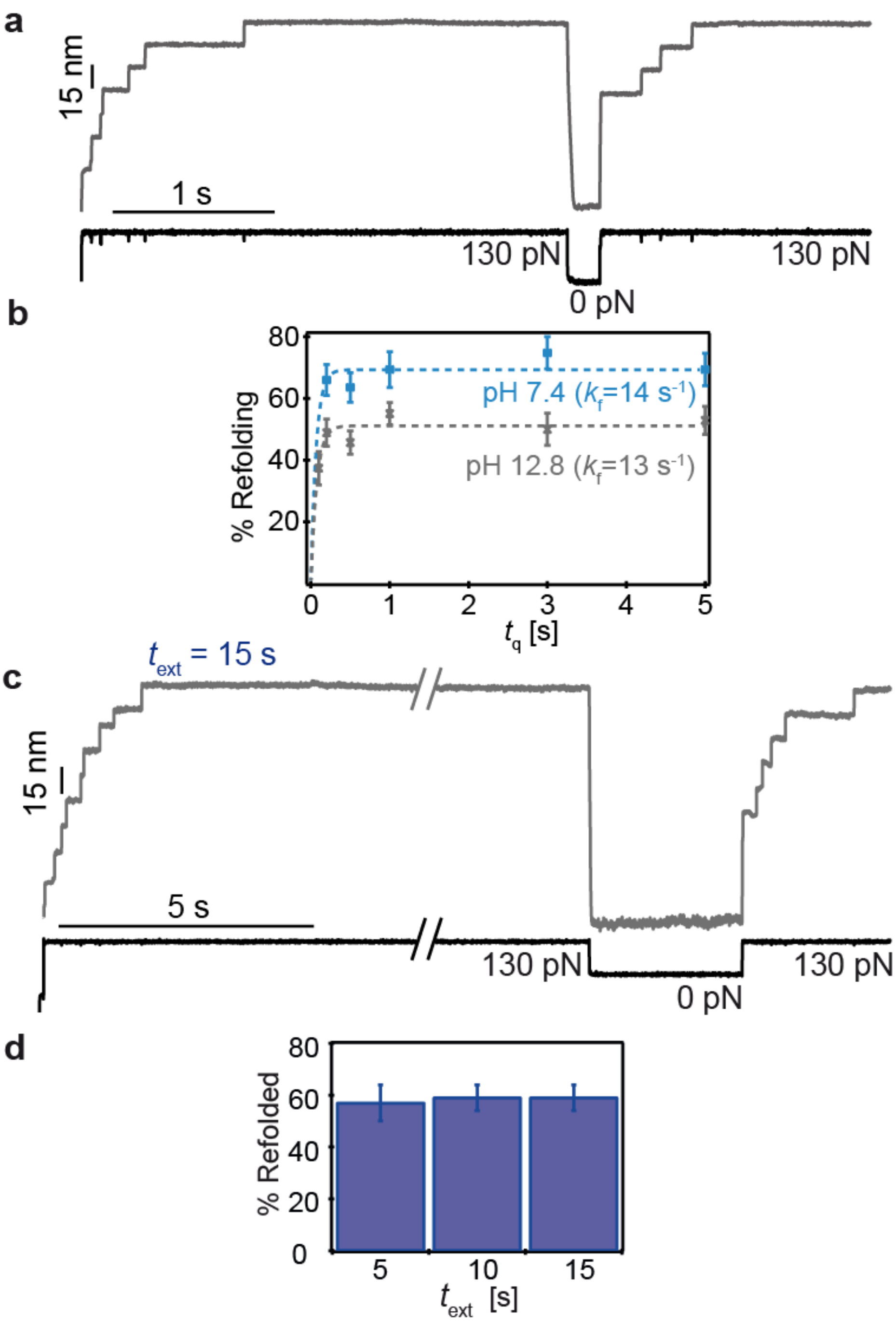


**Supplementary Figure 2. The (I27$_{E24C\text{-}K55C}$)$_8$ polyprotein is able to successfully refold at pH = 12.8 when the disulfide bond remains oxidised.** (**a**) Using a force quench protocol that lacks the high-force pulses employed to trigger disulfide bond reduction, we monitored the refolding of the (I27$_{E24C\text{-}K55C}$)$_8$ polyprotein when it is extended up to the rigid disulfide bond. A first pulse of 130 pN induces the unfolding of the protein up to the disulfide bond, marked by a step-wise increase in length of 15 nm. After 3 seconds, the force is removed for $t_q$ in order to trigger mechanical folding. A *test* pulse back at 130 pN exhibits the re-unfolding of the protein in steps of 15 nm. The recovery of the mechanical stability of the previously unfolded modules unambiguously certifies that the protein has successfully refolded under high pH = 12.8 conditions. (**b**) Measuring the percentage of

successfully refolded modules during the test pulse compared to those occurring in the initial unfolding pulse for distinct quench times ($t_q$) measures the time-course evolution of mechanical folding. Fitting a single exponential fit to the data yields a folding rate $k_f$ = 13 $s^{-1}$ (grey discontinuous line), which closely resembles the folding rate observed in analogous experiments conducted at neutral pH = 7.4, exhibiting a folding rate of $k_f$ = 14 $s^{-1}$(blue discontinuous line). While the experiments conducted at high pH exhibit a slightly lower (19%) efficiency of refolding, probably due to an alkali induced modification of the side chain of any of the amino acids, these results directly demonstrate that $(I27_{E24C-K55C})_8$ polyprotein is able to successfully refold when the disulfide bond has not been reduced under high pH = 12.8 conditions. (**c**) Crucially, the time the protein is left unfolded and extended at high force, $t_{ext}$, does not have any negative impact on the folding efficiency, (**d**), which is kept constant at ~ 58%.

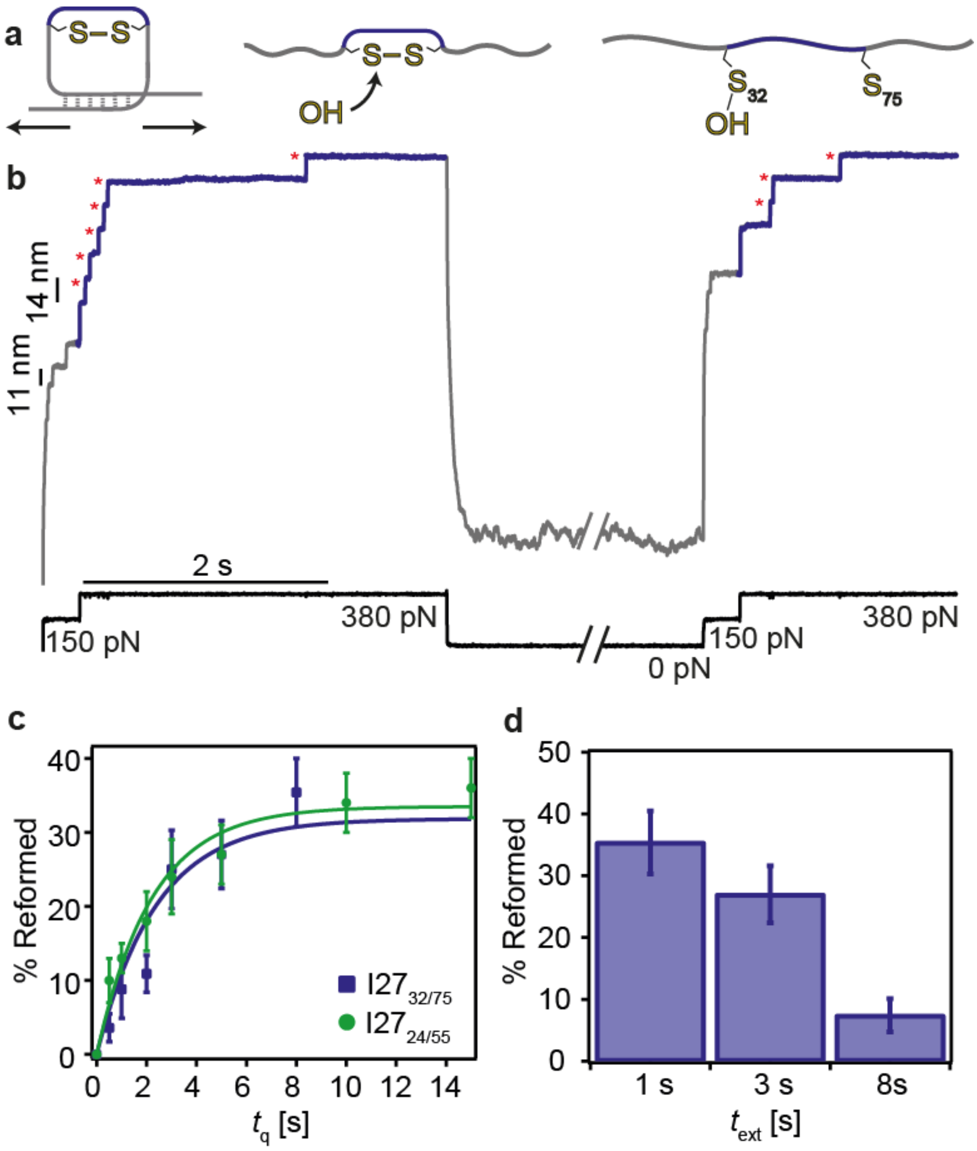


**Supplementary Figure 3. The sulfenic acid–induced reformation of a disulfide bond is independent on the position of the disulfide bond within the protein structure.** (**a**) We repeated the non-enzymatic oxidative folding experiments on the $(I27_{E24C-K55C})_8$ polyprotein described in Fig. 1 in the main text with a similar construct whereby the disulfide bond position is changed, occurring between cysteines 32-75. (**b**) Stretching the resulting $(I27_{G32C–A75C})_8$ polyprotein construct at 150 pN first results in the unfolding of the protein up to the position of the disulfide bond, eliciting steps of 11 nm (grey). A second pulse at a higher force of 380 pN triggers the hydroxyl-induced cleavage of the individual disulfide bonds (red asterisks), fingerprinted by a length increase of 14 nm (purple). (**c**) The percentage of reformed disulfide bonds increases exponentially with the $t_q$ with an associated rate constant of $k_{ref}$ = 0.42 $s^{-1}$ (purple), very similar to that measured for $(I27_{E24C-K55C})_8$ with $k_{ref}$ = 0.45 $s^{-1}$ (green). (**d**) Increasing the exposure time, $t_{ext}$, of the sulfenic acid to the solution, while maintaining $t_q$ = 5 s, decreases the percentage of reformed disulfide bonds, similar to the behaviour observed for $(I27_{E24C-K55C})_8$. These experiments demonstrate that the position of the disulfide bond within the protein structure does not significantly affect the sulfenic acid-mediated mechanism of oxidative folding.

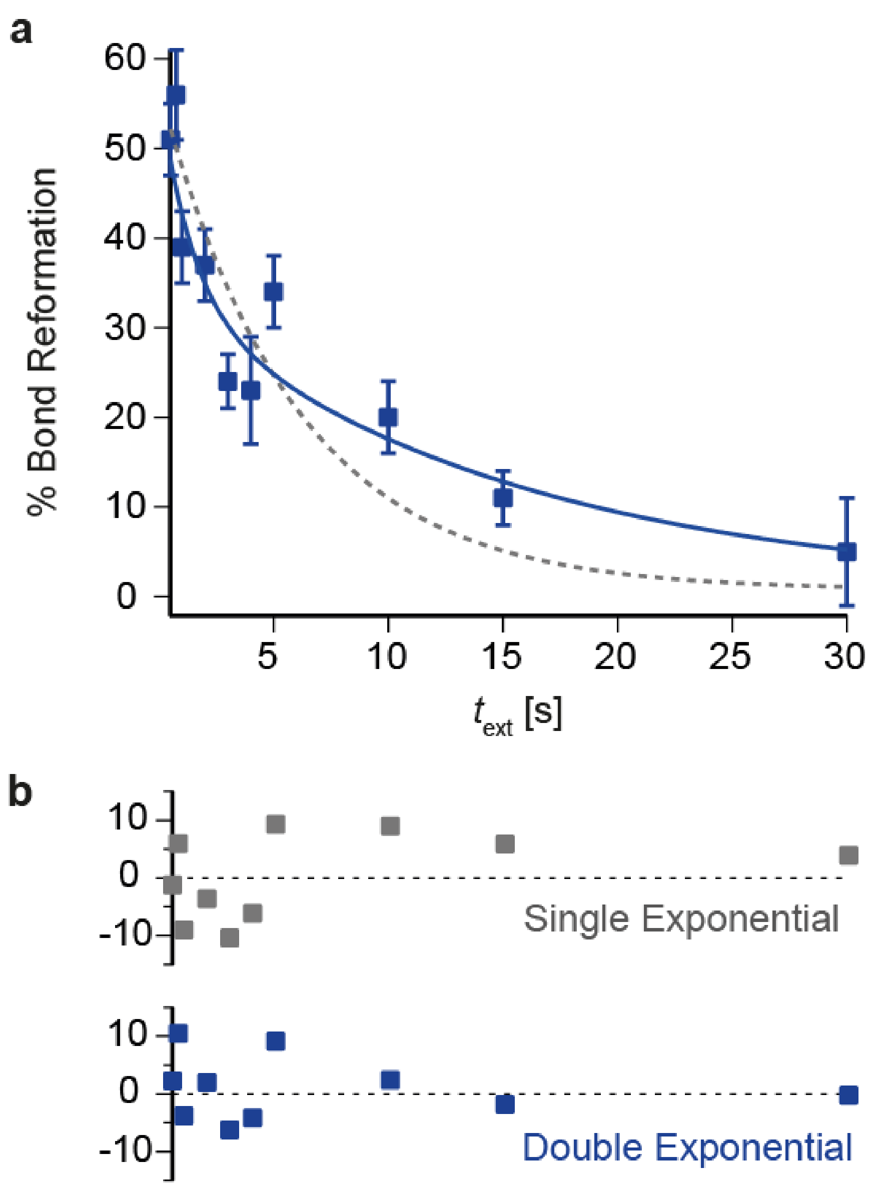


**Supplementary Figure 4. The dramatic decrease of the probability of disulfide reoxidation as a function of $\Delta t_{ext}$ is better captured by a double exponential decay.** (**a**) Fitting the time-course of disulfide bond reformation as a function of the time the reduced protein is kept stretched at a high force, $t_{ext}$, (Figure 3 in the main text) shows a time-course evolution that can be readily captured with a single exponential fit, yielding a decay rate of $k$ =0.17 $s^{-1}$ ($\chi^2_{red}$ = 5.02). However, a double exponential fit, reminiscent of at least two concomitant processes occurring at the same time with decay rates of $k_1$ = 0.065 $s^{-1}$ and $k_2$ = 0.695 $s^{-1}$, fits the data slightly better ($\chi^2_{red}$ = 2.78) as demonstrated in (**b**) in the graphs showing the residuals corresponding to each distinct fit.

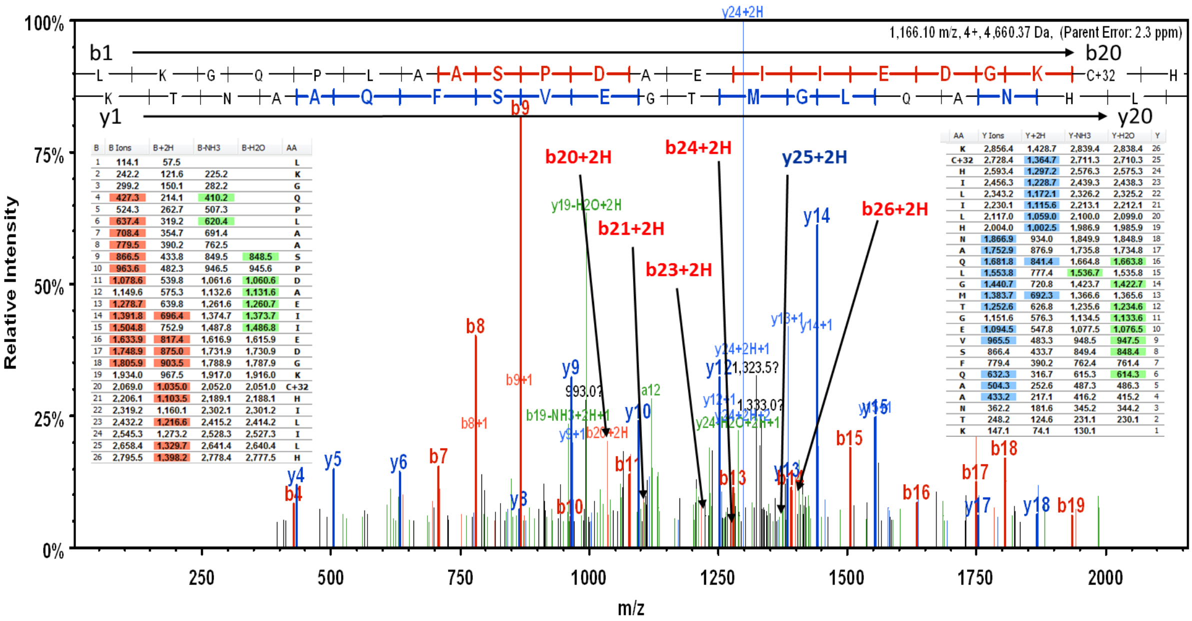


**Supplementary Figure 5. Evidence for sulfinic acid modification on a peptide with *m/z* 1166.10$^{4+}$.** Evidence for a sulfinic acid modification with the addition of 32 Da on the cysteine residue at Cys55. Detection of the modification is found on the doubly-charged b20-ion and correct assignment of residue masses after the modification at the doubly-charged b21, b23, b25 and b26-ions. Assignment of the doubly-charged y25-ion including the modification is further supporting information for the correct assignment.

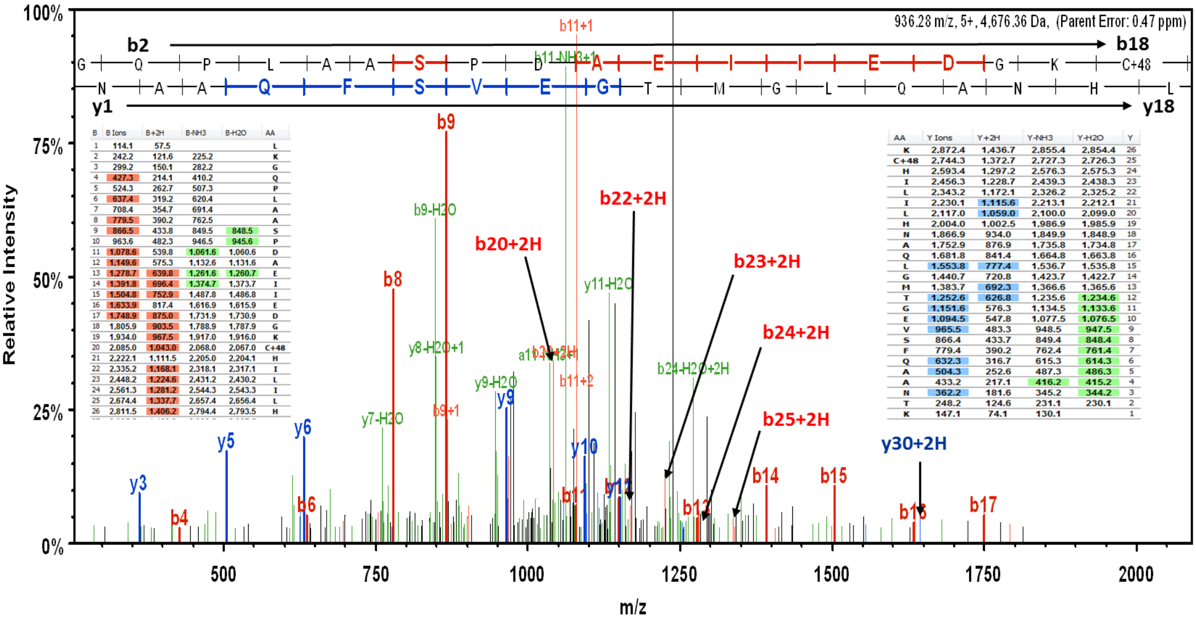


**Supplementary Figure 6. Evidence for a sulfonic acid modification on a peptide with *m/z* 936.28[5+].** Correct peptide identification was provided by the assignment of the sequence data from the b and y-ions in the spectra. The oxidation modification of the cysteine residue, with the addition of 48 Da, was determined by assignment of the doubly-charged b20-ion and subsequent ions after the modification in the peptide sequence (doubly-charged b22 and b25-ions). This determines the modification of sulfonic acid ($SO_3H$) has been correctly assigned to the cysteine residue at Cys55.

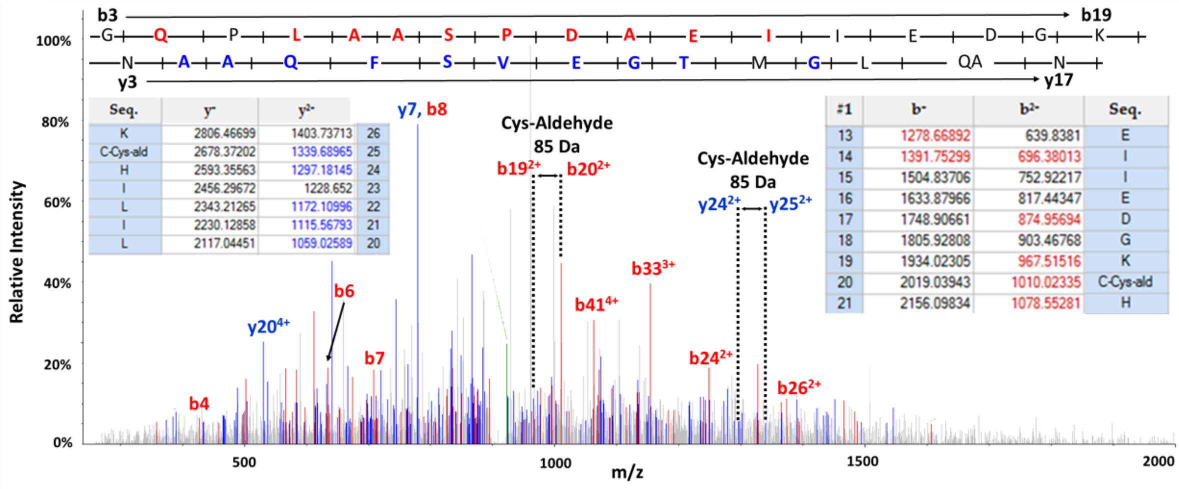


**Supplementary Figure 7. Confirmation of cysteine-aldehyde formation by alpha-elimination in reducing conditions**. A peptide with m/z 923.08$^{5+}$ was detected with a loss of 17.9928 Da from the cysteine residue in the sequence at the doubly-charged b20-ion and corresponding doubly-charged y25-ion. Further evidence was detected in the spectra for correct assignment of the modified cysteine on the y25$^{3+}$, y25$^{4+}$ and y25$^{5+}$ fragment ions as well as the b20$^{3+}$ fragment ion (not shown in the spectra to reduce confusing labelling).

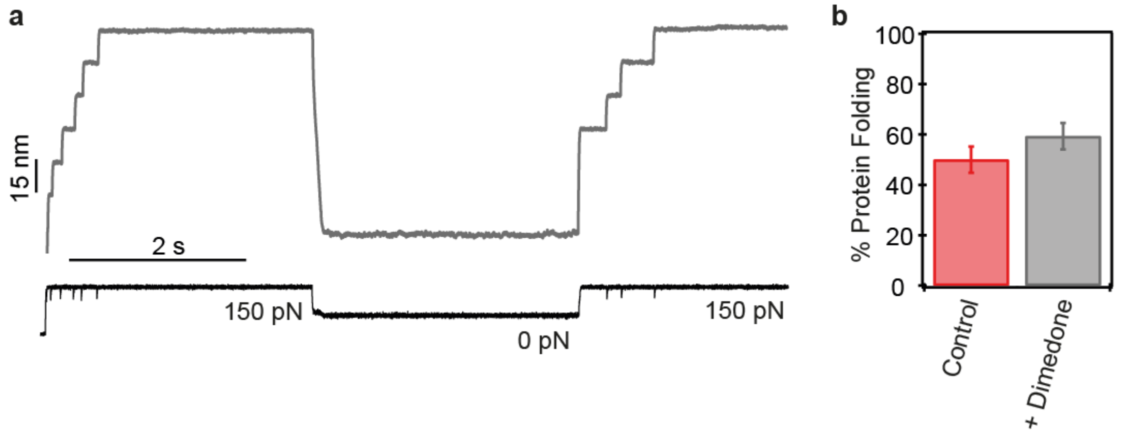


**Supplementary Figure 8. The refolding of the oxidised ($I27_{E24C-K55C}$)$_8$ polyprotein is not blocked by the presence of dimedone.** In order to indisputably prove that dimedone only interacts with the sulfenic acid moiety created upon the alkali-mediated reduction of the disulfide bond, we monitored the folding efficiency of the oxidised ($I27_{E24C-K55C}$)$_8$ polyprotein at pH = 12.8 and under 30 mM of dimedone. As shown in the folding trajectory (**a**) and bar diagram (**b**), dimedone does not impede the successful refolding of the oxidised protein, hallmarked by the presence of 15 nm steps in the *test* pulse. Therefore, these experiments demonstrate that dimedone alone does not interfere with the refolding of the ($I27_{E24C-K55C}$)$_8$ polyprotein.

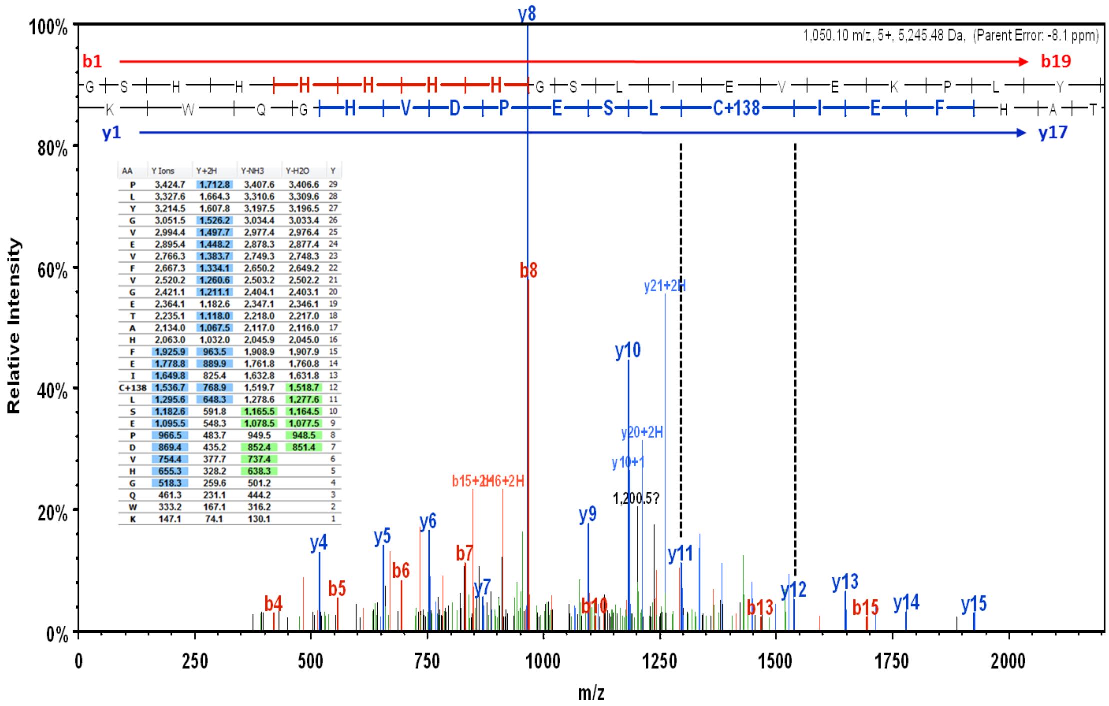


| AA | Y Ions | Y+2H | Y-NH3 | Y-H2O | Y |
|---|---|---|---|---|---|
| P | 3,424.7 | 1,712.8 | 3,407.6 | 3,406.6 | 29 |
| L | 3,327.6 | 1,664.3 | 3,310.6 | 3,309.6 | 28 |
| Y | 3,214.5 | 1,607.8 | 3,197.5 | 3,196.5 | 27 |
| G | 3,051.5 | 1,526.2 | 3,034.4 | 3,033.4 | 26 |
| V | 2,994.4 | 1,497.7 | 2,977.4 | 2,976.4 | 25 |
| E | 2,895.4 | 1,448.2 | 2,878.3 | 2,877.4 | 24 |
| V | 2,766.3 | 1,383.7 | 2,749.3 | 2,748.3 | 23 |
| F | 2,667.3 | 1,334.1 | 2,650.2 | 2,649.2 | 22 |
| V | 2,520.2 | 1,260.6 | 2,503.2 | 2,502.2 | 21 |
| G | 2,421.1 | 1,211.1 | 2,404.1 | 2,403.1 | 20 |
| E | 2,364.1 | 1,182.6 | 2,347.1 | 2,346.1 | 19 |
| T | 2,235.1 | 1,118.0 | 2,218.0 | 2,217.0 | 18 |
| A | 2,134.0 | 1,067.5 | 2,117.0 | 2,116.0 | 17 |
| H | 2,063.0 | 1,032.0 | 2,045.9 | 2,045.0 | 16 |
| F | 1,925.9 | 963.5 | 1,908.9 | 1,907.9 | 15 |
| E | 1,778.8 | 889.9 | 1,761.8 | 1,760.8 | 14 |
| I | 1,649.8 | 825.4 | 1,632.8 | 1,631.8 | 13 |
| C+138 | 1,536.7 | 768.9 | 1,519.7 | 1,518.7 | 12 |
| L | 1,295.6 | 648.3 | 1,278.6 | 1,277.6 | 11 |
| S | 1,182.6 | 591.8 | 1,165.5 | 1,164.5 | 10 |
| E | 1,095.5 | 548.3 | 1,078.5 | 1,077.5 | 9 |
| P | 966.5 | 483.7 | 949.5 | 948.5 | 8 |
| D | 869.4 | 435.2 | 852.4 | 851.4 | 7 |
| V | 754.4 | 377.7 | 737.4 | | 6 |
| H | 655.3 | 328.2 | 638.3 | | 5 |
| G | 518.3 | 259.6 | 501.2 | | 4 |
| Q | 461.3 | 231.1 | 444.2 | | 3 |
| W | 333.2 | 167.1 | 316.2 | | 2 |
| K | 147.1 | 74.1 | 130.1 | | 1 |

**Supplementary Figure 9. Evidence for a modified peptide following exposure to dimedone.** A well matched sequence tag from the y5-y15-ions and from the b5-b8-ions successfully determines the correct assignment of the peptide to the protein of interest including the His tag. A mass shift of *m/z* 138.1 was detected on the cysteine residue at y12. Correct assignment of the subsequent residues following the modified cysteine gives strong evidence for the correct location of the modification.

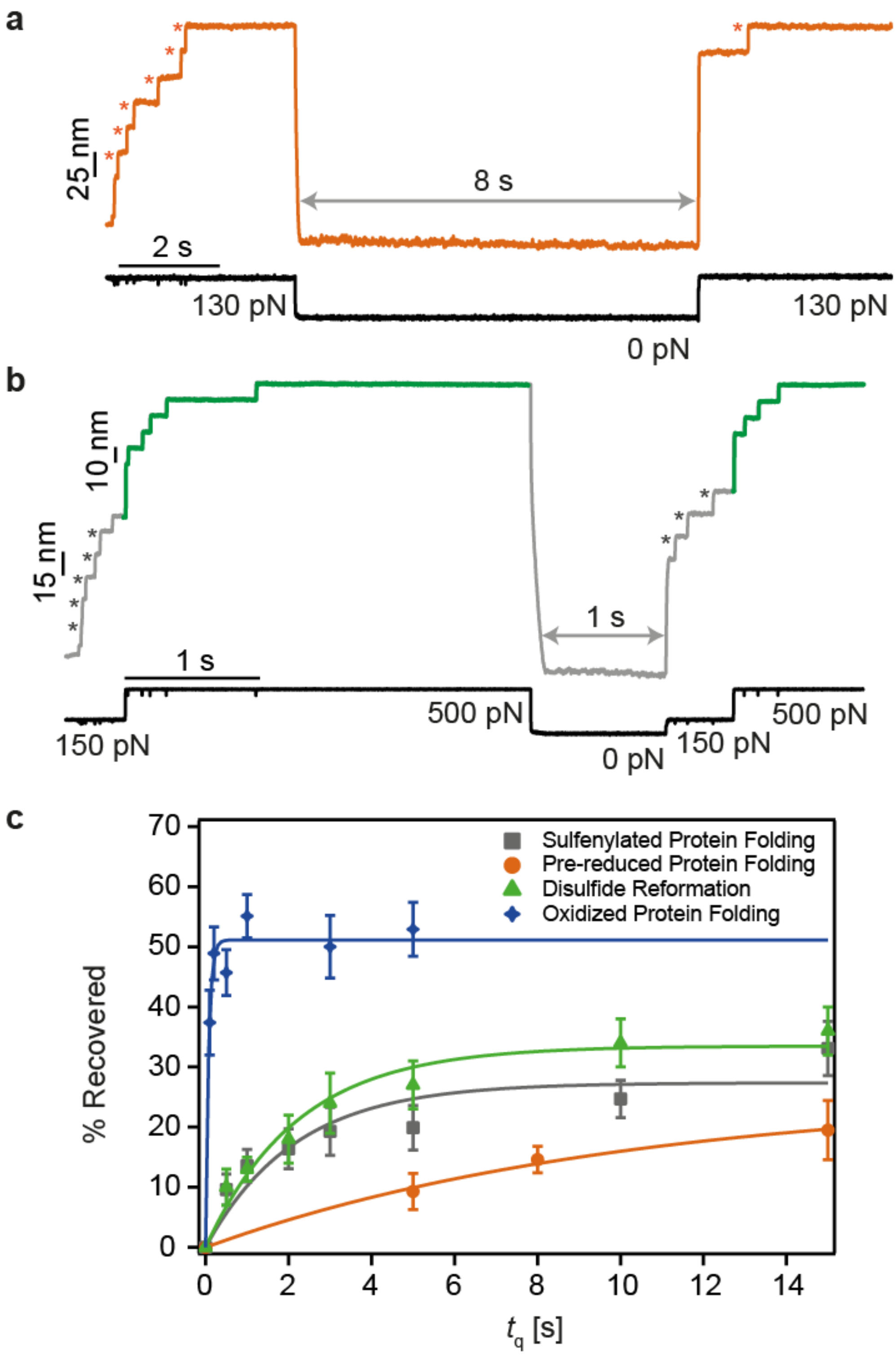


**Supplementary Figure 10. The sulfenic acid-mediated reformation of a disulfide bond increases the rate and yield of protein folding.** (**a**) Incubating $(I27_{E24C\text{-}K55C})_8$ in 10mM Tris(2-carboxyethyl)phosphine hydrochloride (TCEP) for 30 mins prior to AFM experiments results in the full rupture of all disulfide bonds, as fingerprinted by the ~25 nm steps of unfolding, which corresponds to the complete extension of the reduced protein, void of a disulfide bond. The % of refolding of the pre-reduced protein at pH =12.8 is surprisingly low, even at long $t_q$. (**b**) By contrast, after protein sulfenylation (created by the alkaline hydrolysis of

the disulfide bond-containing $(I27_{E24C-K55C})_8$ protein) the refolding efficiency is drastically improved, as observed by the presence of 15 nm steps (grey asterisks) in the test pulse preceding the disulfide bond scission events (green). (**c**) Measuring the percentage of successful refolding events over a range of quench times $t_q$ probes the time-dependent mechanical folding process. Fitting the data with a single exponential yields the associated rate of folding. While the folding kinetics of pre-reduced $(I27_{E24C-K55C})_8$ polyprotein at pH = 12.8 yields a folding rate of $k_{fold}$ = 0.1 $s^{-1}$ (orange), the folding rate of the same sulfenylated protein (grey) increases to up to $k_{fold}$ = 0.46 $s^{-1}$. Crucially, the folding yield is also significantly increased when folding is linked to disulfide bond reformation. For comparison, the kinetics of disulfide bond reformation (green, $k_{ref}$ = 0.45 $s^{-1}$) and oxidised protein (blue, $k_{fold}$ = 13 $s^{-1}$) are shown. Altogether, these results demonstrate that the reformation of the disulfide bond triggered by sulfenic acid increases both the rate and yield of protein refolding, thus acting as a rescue element that prevents the protein from irreversibly misfolding.

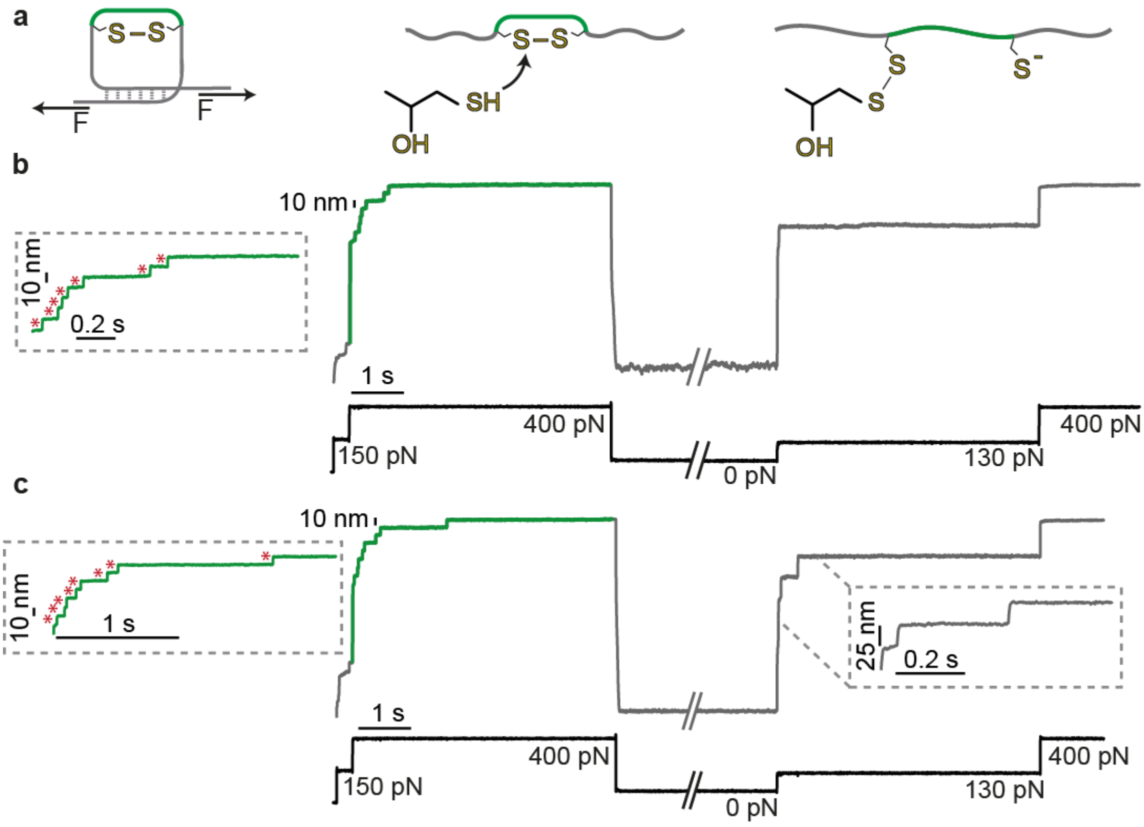


**Supplementary Figure 11. Successful non-enzymatic oxidative folding can only be observed after the presence of an alkali-mediated reduction event, which introduces a sulfenic acid moiety in the protein in a controlled manner.** By contrast, when another nucleophile such as 3-mercapto-1-propanol is used as a disulfide bond reducing agent, disulfide bond cleavage results in the presence of a mixed disulfide and a thiol. Repeating the same force protocol used in Fig.1 in the main text results in trajectories that do not exhibit the reformation of the disulfide bond. (**a**) While in most of the trajectories (~65%) we observed that refolding was blocked (**b**), in the remaining (~35%) of the trajectories at least one 25 nm step was observed (**c**), fingerprinting the correct folding of the protein lacking of a disulfide bond. We hypothesize that this last scenario might correspond to the reduction of the mixed disulfide by another thiol from the solution, leaving the substrate with two free thiols. The presence of the low occurrence 25 nm steps indicates that the reformation of the protein disulfide is discouraged, supporting the idea that two free thiols will not form a disulfide bond unless an oxidant agent (such as sulfenic acid) activates the thiols by catalysing the process. Altogether, these experiments highlight the unique chemical mechanism mediated by sulfenic acid to catalyse the non-enzymatic reformation of the disulfide bond, triggering the successful oxidative folding of the protein.

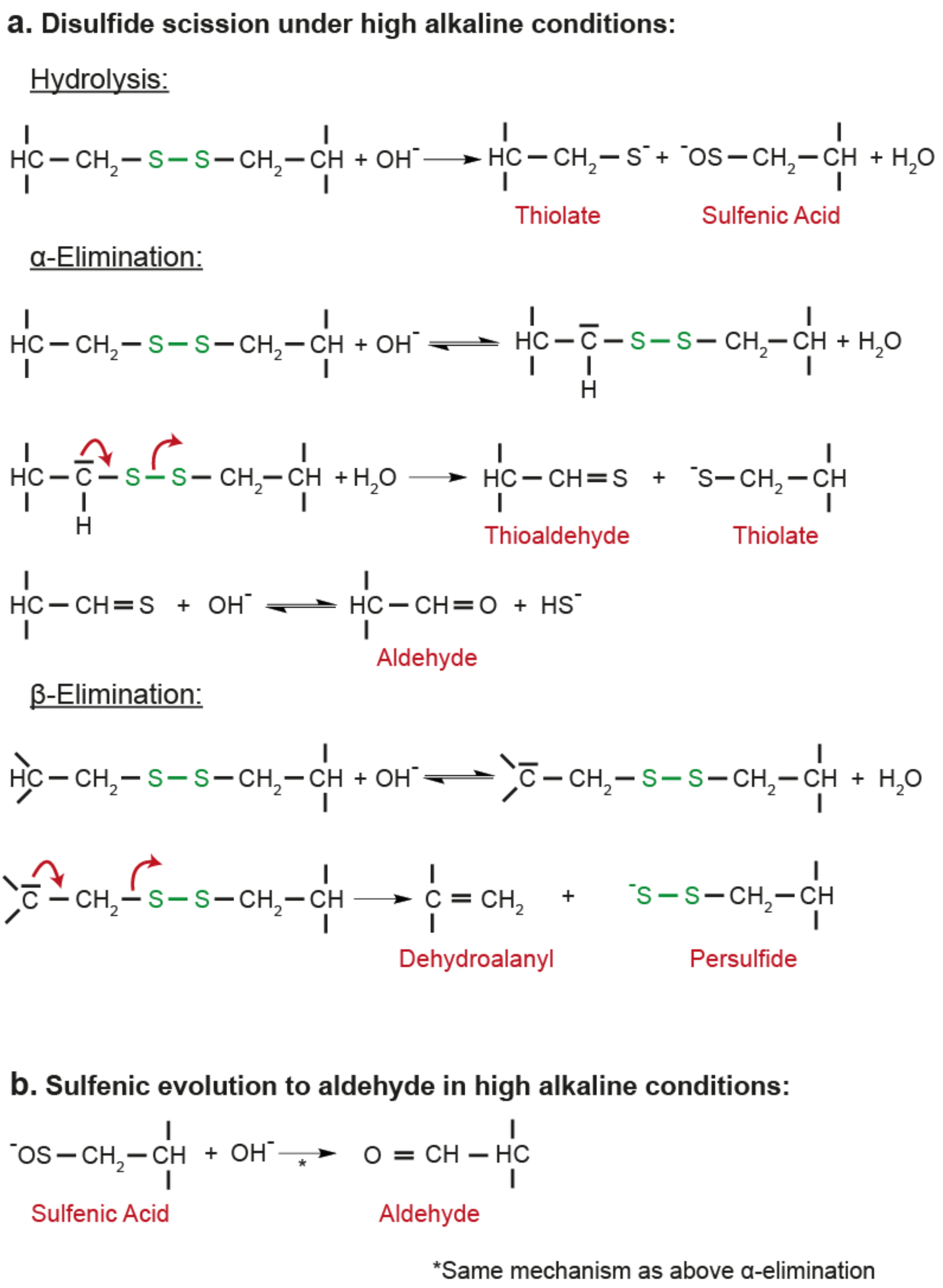


**Supplementary Figure 12. The reactivity of a protein disulfide bond and a sulfenic acid moiety under high alkaline conditions.** (**a**) In solution, three possible documented mechanisms can lead to disulfide bond scission in the presence of hydroxide anions ($OH^-$); (i) The hydrolysis mechanism corresponds to the scission of the S-S bond through an $S_N2$ chemical reaction, giving rise to a sulfenic acid and a thiolate; (ii) In the α-elimination mechanism, the carbon in α position is deprotonated, giving rise to an unstable carbanion that can internally re-attack, giving rise to a thiolate and to a thioaldehyde that rapidly evolves to an aldehyde moiety; (iii) Alternatively, during β-elimination, the carbon in β position

is deprotonated, resulting in a dehydroalanyl and a persulfide. Our MS results show evidences of the presence of sulfenic acid, aldehyde, dehydroalanyl and persulfide (see Supplementary Fig. 7, 13-14 and Table SI1) thus suggesting the presence of the three competing mechanisms. (**b**) Under high pH conditions, the sulfenic acid moiety produced during the hydrolysis of the disulfide bond can evolve into a stable aldehyde moiety via an α-elimination mechanism akin to that observed for the scission of the disulfide bond.

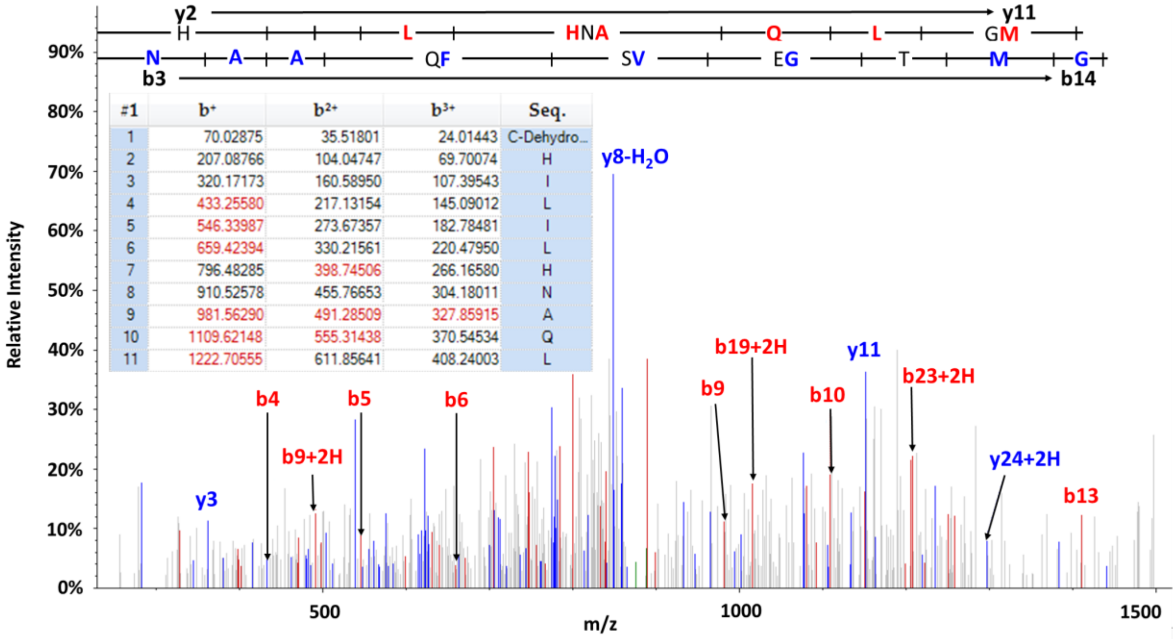


| #1 | b⁺ | $b^{2+}$ | $b^{3+}$ | Seq. |
|---|---|---|---|---|
| 1 | 70.02875 | 35.51801 | 24.01443 | C-Dehydro... |
| 2 | 207.08766 | 104.04747 | 69.70074 | H |
| 3 | 320.17173 | 160.58950 | 107.39543 | I |
| 4 | 433.25580 | 217.13154 | 145.09012 | L |
| 5 | 546.33987 | 273.67357 | 182.78481 | I |
| 6 | 659.42394 | 330.21561 | 220.47950 | L |
| 7 | 796.48285 | 398.74506 | 266.16580 | H |
| 8 | 910.52578 | 455.76653 | 304.18011 | N |
| 9 | 981.56290 | 491.28509 | 327.85915 | A |
| 10 | 1109.62148 | 555.31438 | 370.54534 | Q |
| 11 | 1222.70555 | 611.85641 | 408.24003 | L |

**Supplementary Figure 13. Weak evidence of a dehydroalanine formation from cysteine by beta-elimination in reducing conditions.** A peptide with m/z 888.13$^{3+}$ was detected with a loss of 33.9877 Da from the cysteine residue in the sequence at the singly-charged b4-b6-ions in the fragmentation spectra. The modified residue lies at the b1-ion and was not detected in this spectra along with the b2-ion. The presence of correctly matched b-ions after the loss from the cysteine is evidence to suggest that this modified peptide has been correctly matched in the database. This is weak evidence for the presence of a dehydroalanine from Cys55.

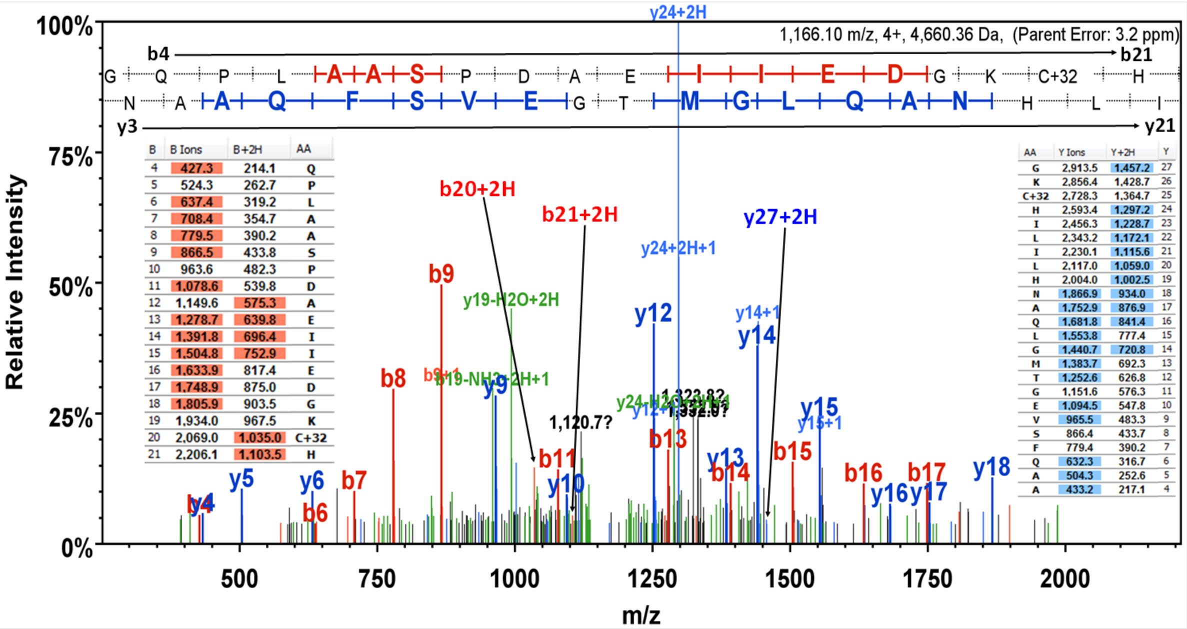


**Supplementary Figure 14. Confirmation of cysteine-sulfide (persulfide) formation in reducing conditions**. A peptide with m/z 1166.09$^{4+}$ was detected with an addition of 31.97207 Da on the cysteine residue in the sequence at the doubly-charged b20-ion. There was no assignment of the modification on the corresponding singly or doubly-charged y25-ion however, correct assignment of the doubly-charged y27-ion after the modification confirms a sulfide modification to the cysteine residue at Cys55.

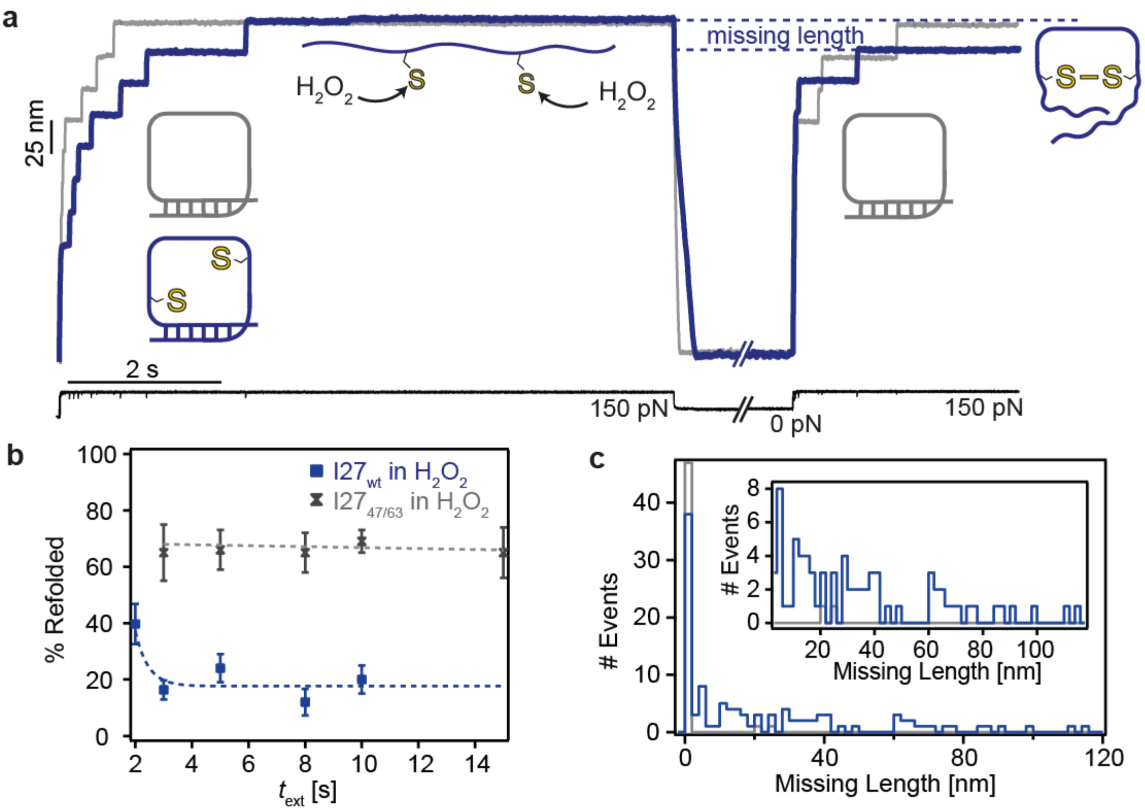


**Supplementary Figure 15. Hydrogen peroxide induces S-sulfenylation in cryptic cysteines and promotes the formation of non-native disulfide bonds in wt-I27.** (**a**) Mechanically unfolding wt-I27 (blue), containing two buried, native cysteines that do not form a disulfide bond, in the presence of 300 μM hydrogen peroxide ($H_2O_2$) gives rise to the regular unfolding pattern where each domain unfolds in steps of ~25 nm. Mechanical unfolding exposes the two previously cryptic cysteines to the oxidative aqueous environment. The force is subsequently quenched down to 0 pN for $t_q$ = 5 s to allow the proteins to refold, before the force is re-applied back again to probe folding success. The folding dynamics of the (wt-I27)$_8$ polyprotein is compared to that of the (I27$_{C47A\text{-}C63A}$)$_8$ polyprotein, devoid of native cysteines (grey). (**b**) Under these oxidative conditions (wt-I27)$_8$ dramatically decreases its folding efficiency, an effect that is further exacerbated with $t_{ext}$. By contrast, the folding success of the (I27$_{C47A\text{-}C63A}$)$_8$ polyprotein is not affected by its exposure to $H_2O_2$. (**c**) A recursive feature of the (wt-I27)$_8$ folding trajectories is its inability to recover its initial unfolded length upon being stretched in the *test* pulse. Such a systematic shortening of the protein, spanning a wide range of lengths, is not observed in the case of the (I27$_{47/63}$)$_8$ polyprotein. These 'missing length' events are suggestive of the formation of non-native disulfide bonds, which increase the stiffness of the protein. Altogether, these experiments demonstrate that cryptic cysteines in the wild-type I27 protein undergo $H_2O_2$-mediated post-translational modifications (S-sulfenylation), which dramatically affect the fate of the mechanical folding efficiency; if sulfenic acid undergoes 'uncontrolled' evolution to hyperoxidised species such as sulfinic and sulfonic acid, a decrease the folding efficiency of the wt-I27 protein is measured. By contrast, upon quenching the force, sulfenic can promote the formation of non-native disulfide bonds that short-cut the protein and diminishes its extensibility.

**Supplementary Table 1**: Identification of cysteine containing modified peptides following exposure to high pH buffer prior to digestion. Mass spectrometry analysis by database searching and manual verification.

| Sequence | Modification | *m/z* | Mr | Charge State | Signal Intensity | Cys Modified |
|---|---|---|---|---|---|---|
| $^{2}$GSHHHHHHGSLIEVEKPLYGVEVFVGETAHFEI**c**LSEPDVHGQWK$^{46}$ | Sulfonic acid* | 1038.69 | 5188.40 | 5$^{+}$ | 44130 | 24 |
| $^{49}$GQPLAASPDAEIIEDGK**c**HILILHNAQLGMTGEVSFQAANTK$^{90}$ | Sulfinic acid* | 1118.05 | 4468.16 | 4$^{+}$ | 31880 | 55 |
| $^{49}$GQPLAASPDAEIIEDGK**c**HILILHNAQLGMTGEVSFQAANTK$^{90}$ | Sulfonic acid | 1122.04 | 4484.15 | 4$^{+}$ | 6809 | 55 |
| $^{66}$**c**HILILHNAQLGMTGEVSFQAANTK$^{90}$ | Dehydroalanine* | 888.13 | 2662.38 | 3$^{+}$ | 40950 | 55 |
| $^{47}$LKGQPLAASPDAEIIEDGK**c**HILILHNAQLGMTGEVSFQAANTK$^{90}$ | Aldehyde | 923.08 | 4611.39 | 5$^{+}$ | 8260 | 55 |
| $^{47}$LKGQPLAASPDAEIIEDGK**c**HILILHNAQLGMTGEVSFQAANTK$^{90}$ | Persulfide | 1166.09 | 4661.34 | 4$^{+}$ | 83530 | 55 |
| $^{47}$LKGQPLAASPDAEIIEDGK**c**HILILHNAQLGMTGEVSFQAANTK$^{90}$ | Sulfinic acid | 1166.10 | 4660.37 | 4$^{+}$ | 49590 | 55 |
| $^{47}$LKGQPLAASPDAEIIEDGK**c**HILILHNAQLGMTGEVSFQAANTK$^{90}$ | Sulfonic acid | 1170.09 | 4676.33 | 4$^{+}$ | 21760 | 55 |

*weak evidence

**Supplementary Table 2**: Cysteine containing peptides modified with Sulfenic acid-dimedone following database searching and manual verification.

| Sequence | Modification | *m/z* | Mr | Charge State | Signal Intensity | Cys Modified |
|---|---|---|---|---|---|---|
| $^{2}$GSHHHHHHGSLIEVEKPLYGVEVFVGETAHFEI**c**LSEPDVHGQWK$^{46}$ | Dimedone | 1050.10 | 5245.48 | 5$^{+}$ | 92030 | 24 |
| $^{47}$LKGQPLAASPDAEIIEDGK**c**HILILHNAQLGMTGEVSFQAANTK$^{90}$ | Dimedone | 1192.62 | 4766.47 | 4$^{+}$ | 12570 | 55 |
| $^{49}$GQPLAASPDAEIIEDGK**c**HILILHNAQLGMTGEVSFQAANTK$^{90}$ | Dimedone | 1132.33 | 4525.27 | 4$^{+}$ | 114800 | 55 |
| $^{66}$**c**HILILHNAQLGMTGEVSFQAANTK$^{90}$ | Dimedone | 709.36 | 2833.43 | 4$^{+}$ | 13730 | 55 |
